# Enzymes and microorganisms jointly promote the fermentation of rapeseed cake

**DOI:** 10.3389/fnut.2022.989410

**Published:** 2022-09-15

**Authors:** Yujie Song, Litao Sun, Shuning Zhang, Kai Fan, Huan Wang, Yujie Shi, Yaozong Shen, Wenmei Wang, Jie Zhang, Xiao Han, Yilin Mao, Yu Wang, Zhaotang Ding

**Affiliations:** ^1^Tea Research Institute, Qingdao Agricultural University, Qingdao, China; ^2^Tea Research Institute, Shandong Academy of Agricultural Sciences, Jinan, China; ^3^Co-construction Service Center of Three Districts in Taolin Town, Shandong, China

**Keywords:** rapeseed cake, enzymatic fermentation, bacillus, *Lysinibacillus*, organic acid, amino acid, fatty acid

## Abstract

Rapeseed cake is a by-product of rapeseed oil separation. The nutritional components of rapeseed cake mainly include a variety of carbohydrates, proteins, and minerals. In order to improve the conversion rate of rapeseed cake, we studied the physicochemical properties, the structure of microbial communities, and the composition of metabolites in rapeseed cake after enzymatic fermentation. The results showed that the addition of enzymatic preparation increased microbial diversity. The relative abundance of *Bacillus*, *Lysinibacillus*, *Empedobacter*, *Debaryomyces*, *Hyphopichia*, and *Komagataella* in enzymatic fermentation was significantly higher than that in natural fermentation. Unlike natural fermentation, microbial diversity during enzymatic fermentation is specific, which improves the efficiency of fermentation. Otherwise, enzymatic fermentation promotes the conversion of macromolecular substances in rapeseed cake, which increases small metabolites, such as fatty acids, organic acids, amino acids and their derivatives. The metabolite enrichment pathway is mostly concentrated in sugar metabolism and fatty acid metabolism. In conclusion, after adding enzymatic preparation, enzymes and microorganisms jointly promote the transformation of macromolecules during the fermentation of rapeseed cake, which laid a good foundation for further utilization of rapeseed cake.

## Introduction

Rapeseed cake is a by-product of rapeseed oil separation. It has a huge output in China. It is mainly composed of protein, wood fiber, and minerals (1). Its crude protein content is 31–35.7%, and the crude protein contains 82% amino acids, with a good balance of essential amino acids and a relatively high content of sulfur-containing amino acids (2). Usually, the rapeseed cake is used as animal feedstuffs or soil fertilizers (3).

Rapeseed cake is a feed component with huge economic potential. However, it contains anti-nutritional compounds, such as glucosinolates, phytic acid, alkaloids, erucic acid, and tannins, which may be harmful to the digestive system of animals (4). Therefore, the direct use of rapeseed cake as animal feed is greatly limited. In order to improve the effective utilization of rapeseed cake, it needs to be fermented. Currently, the results showed that the solid-state fermentation of rapeseed cake by *Aspergillus niger* was helpful to degrade glucosinolates, improve their nutritional value, and improve their application in animal feed (2). In the process of solid-state fermentation, inoculation of the fungus *Mortierella alpina* has the potential to improve the nutritional value of rapeseed cake. At the same time, the utilization of linseed oil as a supplement to the fermentation was positive for fungal growth and the production of protein (5). During solid-state fermentation, adding mixed fungi such as *Rhyzopus oryzae*, *Aspergillus ibericus*, and *Aspergillus niger* can obtain more cellulase and xylanase, which can improve bioactive compounds, so as to improve the nutritional value of rapeseed cake and improve animal feed by-products (6).

Rapeseed cake is also a traditional organic fertilizer widely used in crops, vegetables, fruit trees, etc. It has been shown that the application of rapeseed cake fertilizer in rice fields could increase the soil microbial population and improve the rice yield by regulating the nutrient retention and release process (7–9). Mixing rapeseed cake and inorganic fertilizer is beneficial to increasing corn plant biomass and improving crop productivity, soil fertility, and nutrient balance (10, 11). However, as a kind of fertilizer, rapeseed cake is mainly applied directly, which is difficult to give full play to its best fertilizer efficiency, resulting in the shortcomings of high cost and slow fertilizer effect.

In order to improve the utilization of rapeseed cake, the changes in nutrients, microbial community structure, and metabolites of rapeseed cake during natural fermentation and enzymatic fermentation were studied, which provided a theoretical basis for the fermentation and deep utilization of rapeseed cake.

## Materials and methods

### Fermentation and collection of rapeseed cake

The experiment was conducted in Qingdao, Shandong Province, China (36° 18′ N, 120° 07′ E) in 2021. All the rapeseed cake in this test were purchased from Xincang’s No.1 oil manufacture (Anhui, China). The appearance of the rapeseed cakes was irregular and fragmented, and the color was brown to dark brown, with the aroma of cooked rapeseed. The fermentation barrel is made of high-density polyethylene, with a capacity of 25 L and good sealing performance. The high-efficiency enzymatic preparation is a mixture powder with bacteria and enzymes. Its main components are lipase, hemicellulase, yeast, lactic acid bacteria, amylolytic bacillus, and other digestive bacteria. The number of effective viable bacteria is ≥ 20 billion/g, and it is easy to synthesize digestive enzymes, with total enzyme activity > 10,000 μ/g.

The experiment was divided into two groups: natural fermentation (T1) and enzymatic fermentation (T2), with 3 fermentation barrels in each group. Add 8 kg rapeseed cake and 16 kg distilled water into each fermentation barrel, and the feed water ratio is 1:2. In T2, the high-efficiency enzymatic preparation shall be added in rapeseed cake at a 3‰ ratio based on the total weight of rapeseed cake and water. Then, evenly mixed and sealed. The fermentation process is facultative anaerobic until the fermentation is complete. Random sampling was carried out for each treatment in the four stages: the non-fermented stage (0d, S0), early stage (3d, S1), middle stage (7d, S2), and late stage (14d, S3). Then, each sample with 3 replications was divided into two parts, one part was stored at 4°C to determine its nutritional properties, and the other part was frozen at –80°C to determine its microbial composition and metabolites.

### Determination of nutritional indicators

The contents of the free amino acid (AA) and the free fatty acids (FFA) were determined by using Suzhou Grace Biotechnology kits (China) following the manufacturer’s instructions. The total nitrogen (TN) content was analyzed by the Kjeldahl method, total phosphorus (TP) content was analyzed by the AutoAnalyzer system, and the total potassium (TK) content was analyzed by atomic absorption spectrophotometer (12). The content of organic carbon was analyzed by the K_2_Cr_2_O_7_/H_2_SO_4_ method (13). According to the method of Ortiz et al., the contents of mineral elements were determined (14). The enzymatic activities of lipase, hemicellulase, α-amylase, phytase, polygalacturonase, and cellulase were determined using microplate assay kits (Suzhou Grace Biotechnology Co., Ltd.).

### Determination of microbiomes

#### Extraction and polymerase chain reaction amplification of genomic deoxyribonucleic acid

According to the manufacturer’s protocols, the genomic deoxyribonucleic acid (DNA) of the rapeseed cake was extracted by CTAB method (15–17). Then, the purity and concentration of DNA were detected by agarose gel electrophoresis. A suitable sample of DNA was used in the centrifuge tube, and the sample was diluted with sterile water to 1 ng/μL. Using the diluted genomic DNA as the template. The DNA samples were individually amplified in V3 + V4 hyper variable regions by polymerase chain reaction (PCR) using primers 341F (5′-CCTAYGGGRBGCASCAG- 3′) and 806R (5′-GGACTACNNGGGTATCTAAT- 3′) for 16S rDNA in bacteria, and primers ITS5-1737F (5′- GGAAGTAAAAGTCGTAACAAGG- 3′) and ITS2-2043R (5′- GCTGCGTTCTTCATCGATGC- 3′) for ITS in fungi. Phusion ^®^ High fidelity PCR Master Mix with GC buffer (New England Biolabs, United States) and high-efficiency and high-fidelity enzyme for PCR to ensure amplification efficiency and accuracy. PCR was carried out under the following conditions: 98°C for 30 s, 50°C for 30 s, and 72°C for 30 s for a total of 30 cycles. Then, the resulting PCR product was extracted from a 2% agarose gel and further purified and recycled using the Universal DNA purification kit (TianGen, China).

#### Illumina sequencing and processing of sequencing data

The purified amplicons were pooled on the Illumina NovaSeq platform (Illumina, San Diego, CA, United States) of equal molecular weight and double-end sequencing according to the standard protocol of MetWare Biotechnology Co., Ltd. (Wuhan, China). The sheared original sequencing sequences were merged by FLASH software (V1.2.7).^1^ Refer to Qiime (V1.9.1)^2^ Tags quality control process to filter the quality of the connection sequence. Then the sequences were compared with the reference database using the VSEARCH algorithm^3^ to detect chimeric sequences, and then the chimeric sequences were removed. The effective sequences were used in the final analysis. Sequences were grouped into operational taxonomic units (OTUs) at 97% sequence identity by using the clustering algorithm UPARSE (V7.0.1001).^4^ Using the confidence threshold of 70%, the classification of each 16S rRNA gene sequence was analyzed against the Silva database (SILVA138)^5^ through the Mothur classifier algorithm. Using the confidence threshold of 70%, the classification of each ITS sequence was analyzed against the Unite database (V 8.2)^6^ through the Blast^7^ classifier algorithm. Fast multi-sequence alignment was performed using MUSCLE (V3.8.31)^8^ software, finally, the data of each sample were homogenized.

### Determination of metabolomics

The total metabolic profiles of the rapeseed cake with fermentation were performed using two independent approaches: UPLC-MS/MS and GC-MS. Metabolome analysis was performed by MetWare Biotechnology Co., Ltd. (Wuhan, China).

For UPLC-MS/MS analysis, extraction procedure and UPLC-MS/MS procedure were performed as described by Fraga et al. (18) and Chen et al. (19). For GC- MS analysis, the frozen samples of rapeseed cake were ground to a powder in liquid nitrogen. 1 g of the powder was transferred immediately to a 20 mL head-space vial (Agilent, Palo Alto, CA, United States), containing NaCl saturated solution, to inhibit any enzyme reaction. The vials were sealed using crimp-top caps with TFE-silicone headspace septa (Agilent, United States). The GC-MS procedure was modified based on previous studies (20). Briefly, the identification and quantification of derivatized samples were carried out using an Agilent Model 8890 GC and a 5977B mass spectrometer (Agilent, United States), equipped with a DB-5MS capillary column (5% phenyl-polymethyl siloxane: 30 m × 0.25 mm × 0.25 μm). Helium was used as the carrier gas at a linear velocity of 1.2 mL/min. The injector temperature was kept at 250°C and the detector at 280°C. The oven temperature was programmed from 40°C (3.5 min), increasing at 10°C/min to 100°C, at 7°C/min to 180°C, at 25°C/min to 280°C, and hold for 5 min. Mass spectra was recorded in electron impact (EI) ionization mode at 70 eV. The quadrupole mass detector, ion source, and transfer line temperatures were set, respectively, at 150°C, 230°C and 280°C. Mass spectra was scanned in the range m/z 50–500 amu at 1 s intervals. Identification of volatile compounds was achieved by comparing the mass spectra with the data system library (MWGC or NIST) and linear retention index.

### Metabolite analysis

The HCA (hierarchical cluster analysis) results of samples and metabolites were presented as heatmaps with dendrograms, while Pearson correlation coefficients (PCC) between samples were calculated by the core function in R and presented as only heatmaps. Both HCA and PCC were carried out by R package ComplexHeatmap. For HCA, normalized signal intensities of metabolites (unit variance scaling) are visualized as a color spectrum. Significantly regulated metabolites between groups were determined by VIP ≥ 1 and absolute log2FC (fold change) ≥ 1. VIP values were extracted from OPLS-DA result, which also contains score plots and permutation plots, and were generated using the R package MetaboAnalystR. The data was log transform (log2) and mean centering before OPLS-DA. In order to avoid overfitting, a permutation test (200 permutations) was performed.

### Kyoto encyclopedia of genes and genomes annotation and enrichment analysis

Identified metabolites were annotated using the kyoto encyclopedia of genes and genomes (KEGG) Compound database^9^, and annotated metabolites were then mapped to the KEGG Pathway database.^10^ Pathways with significantly regulated metabolites mapped to were then fed into MSEA (metabolite sets enrichment analysis), and their significance was determined by the hypergeometric test’s *p*-values.

### Statistical analysis

Statistical analysis was performed using SPSS 20.0 software (SPSS Inc., Chicago, United States). One-way analysis of variance (ANOVA) and Duncan’s test were used to determine significant differences (*p* < 0.05) among rapeseed cake nutrition analysis (GraphPad Prism 8.0) and the relative abundance of microbial taxa. Alpha diversity indexes were calculated by Qiime (Version 1.9.1), and the difference between beta diversity index groups was analyzed using R (Version 2.15.3). Spearman correlation coefficient (*R*^2^ ≥ 0.64 and *p* ≤ 0.05) was also used to analyze the correlation between the composition of microbial communities and metabolites in the rhizosphere.

## Results

### Effects of different fermentation methods and stages on the nutritional components of rapeseed cake

**FIGURE 1:**
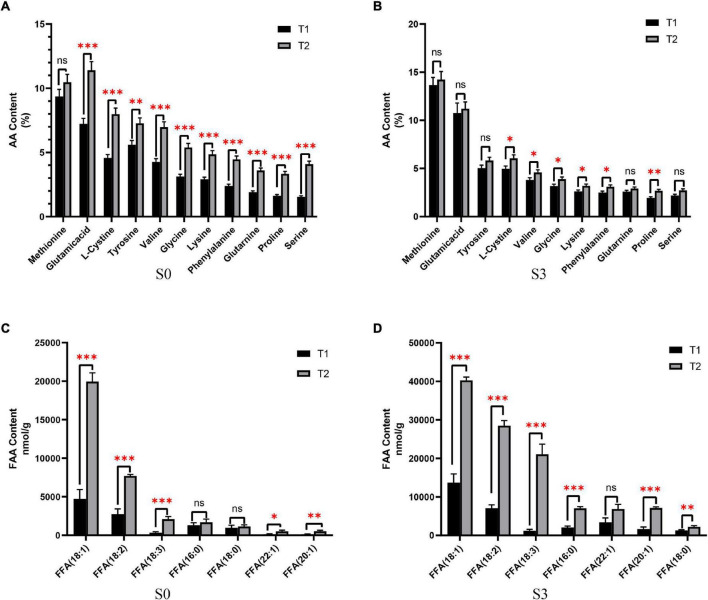
The content of amino acids (AA) and free fatty acids (FFA). **(A)** AA content of fermented rapeseed cake in S1. **(B)** AA content of fermented rapeseed cake in S3. **(C)** FFA content of fermented rapeseed cake in S1. **(D)** FFA content of fermented rapeseed cake in S3. *p < 0.05; **p < 0.01; ***p < 0.001.

In order to study the nutritional status of rapeseed cake under fermentation, and determine the degree of fermentation in rapeseed cake, the physical and chemical indexes of different stages were analyzed (Table 1). The results showed that the contents of AA and FFA increased gradually during the whole fermentation process. Compared with the S0, the S3 of T1 increased by 88.6 and 75.2% respectively, and the S3 of T2 increased by 93.1% and 2.13 times, respectively. In order to further study the composition of amino acids and fatty acids in rapeseed cake after fermentation, we analyzed the contents of 20 amino acids and fatty acids in four stages. The results showed that the content of methionine in T1 and T2 increased significantly with continuous fermentation, while the contents of tyrosine, valine, and lysine decreased. In S3, there were significant differences in some amino acids between the T1 and T2 (*p* < 0.05) (Figures 1A,B). With the continuous fermentation, oleic acid (FFA18:1), linoleic acid (FFA18:2), linolenic acid (FFA18:3), palmitic acid (FFA16:0), erucic acid (FFA22:1), eicosapentaenoic acid (FFA20:1) and stearic acid (FFA18:0) increased significantly. In S3, except erucic acid (FFA22:1), other components had extremely significant differences in T1 and T2 (p < 0.01), and the contents in T2 were higher than in T1 (Figures 1C,D).

**TABLE 1:** Physical and chemical properties of T1 and T2 in fermented rapeseed cake in four stages.

Stage	Sample	pH	AA (μ mol/g)	FFA (μ mol/g)	OM (%)	TN (%)	TP (%)	TK (%)
S0	T1	4.27 ± 0.06a	2.97 ± 0.37a	1.13 ± 0.33b	75.01 ± 2.19b	6.51 ± 0.21a	1.04 ± 0.03a	0.99 ± 0.03a
	T2	4.26 ± 0.09a	2.88 ± 0.66a	2.97 ± 0.54a	82.96 ± 8.79a	6.56 ± 0.02a	1.03 ± 0.02a	0.95 ± 0.04a
S1	T1	4.39 ± 0.16b	3.36 ± 0.71a	1.24 ± 0.43b	73.97 ± 3.09b	7.01 ± 0.06b	0.91 ± 0.01a	0.62 ± 0.02a
	T2	4.59 ± 0.14a	3.35 ± 0.55a	7.07 ± 0.35a	75.12 ± 4.27a	8.10 ± 0.85a	0.85 ± 0.12b	0.66 ± 0.04a
S2	T1	4.41 ± 0.10b	4.37 ± 0.26a	1.32 ± 0.29b	75.59 ± 4.44a	7.09 ± 0.21b	0.76 ± 0.04b	0.69 ± 0.03b
	T2	4.66 ± 0.12a	4.61 ± 0.40a	8.28 ± 0.30a	76.06 ± 2.65a	7.33 ± 0.07a	1.06 ± 0.11a	0.86 ± 0.11a
S3	T1	4.43 ± 0.18a	5.60 ± 1.14a	1.98 ± 0.45b	76.78 ± 1.44a	7.04 ± 0.11b	0.74 ± 0.02b	0.56 ± 0.02b
	T2	4.49 ± 0.18a	5.65 ± 0.32a	9.30 ± 0.82a	75.91 ± 1.97a	7.39 ± 0.04a	1.12 ± 0.06a	0.76 ± 0.11a

The different letters have significant differences (*p* < 0.05).

During the whole fermentation process, the content of OM tended to be stable without obvious change. In S3, the contents of total nitrogen, total phosphorus, and total potassium in T2 were significantly higher than those in T1 (*p* < 0.05). In addition, the mineral elements were analyzed in the fermented rapeseed cake, and there was no significant difference between enzymatic fermentation and natural fermentation (Supplementary Table 1). It indicated that the enzymatic preparation did not affect the content of trace elements.

### Effects of different fermentation methods and stages on microbial community composition in rapeseed cake

To further clarify the changes in microbial community composition in rapeseed cake during fermentation, 16sDNA and ITS of microorganisms in rapeseed cake were sequenced. We used the alpha diversity indexes to judge the richness of microbial communities. During the whole fermentation process, the diversity coverage of bacteria and fungi exceeded 99.9%, indicating that the sequencing data were sufficient and the microbial community was almost covered. For bacteria, the diversity of T1 was significantly higher than that of T2 in S1 and S2 (*p* < 0.05), while the bacterial diversity of T2 was significantly higher than that of T1 in S3 (*p* < 0.05). For fungi, the diversity of T2 was higher than that of T1 in S0 and S1 (*p* < 0.05), while there was no significant difference between the two groups in S3. Chao1 index was used to calculate the abundance of microorganisms. There was no significant difference in abundance between the two groups at all fermentation stages (Table 2).

**TABLE 2:** T1 and T2 alpha diversity index in fermented rapeseed cake in four stages.

Stage	Sample	Shannon		Simpson		Chao1		Ace		Coverage	
		Bacteria	Fungi	Bacteria	Fungi	Bacteria	Fungi	Bacteria	Fungi	Bacteria	Fungi
S0	T1	3.01 ± 0.65a	2.62 ± 0.38b	0.73 ± 0.08a	0.61 ± 0.10b	400.60 ± 94.10a	257.65 ± 50.5a	436.20 ± 108.49a	265.31 ± 48.3a	0.998	0.998
	T2	2.66 ± 0.39b	3.09 ± 0.60a	0.60 ± 0.09b	0.77 ± 0.08a	370.48 ± 5.44a	160.66 ± 17.1a	381.93 ± 19.86b	164.53 ± 19.6a	0.998	0.999
S1	T1	4.89 ± 0.18a	3.92 ± 0.41b	0.90 ± 0.02a	0.84 ± 0.06a	329.48 ± 10.79a	402.47 ± 90.6a	340.20 ± 3.16a	247.03 ± 92.2b	0.997	9.997
	T2	3.66 ± 0.09b	5.02 ± 0.73a	0.78 ± 0.01b	0.92 ± 0.03a	306.75 ± 9.42a	249.41 ± 36.9b	314.28 ± 6.71a	339.35 ± 25.9a	0.999	0.997
S2	T1	5.31 ± 0.29b	3.31 ± 0.29a	0.94 ± 0.02b	0.82 ± 0.04a	392.71 ± 26.91a	285.10 ± 61.3a	397.05 ± 27.26a	309.32 ± 51.5a	0.999	0.998
	T2	4.89 ± 0.19b	2.93 ± 0.39b	0.93 ± 0.01a	0.77 ± 0.07b	348.59 ± 24.29a	255.09 ± 95.7a	356.88 ± 15.93b	258.69 ± 98.7a	0.997	0.998
S3	T1	4.41 ± 0.19b	2.11 ± 0.32a	0.86 ± 0.01b	0.56 ± 0.11a	366.09 ± 17.14a	267.33 ± 49.0a	374.47 ± 20.70a	275.52 ± 46.1a	0.999	0.998
	T2	5.36 ± 0.19a	1.75 ± 0.78a	0.94 ± 0.01a	0.40 ± 0.19a	334.77 ± 25.27a	200.82 ± 55.6a	342.50 ± 23.24a	217.52 ± 68.9a	0.998	0.999

The different letters have significant differences (*p* < 0.05).

**FIGURE 2:**
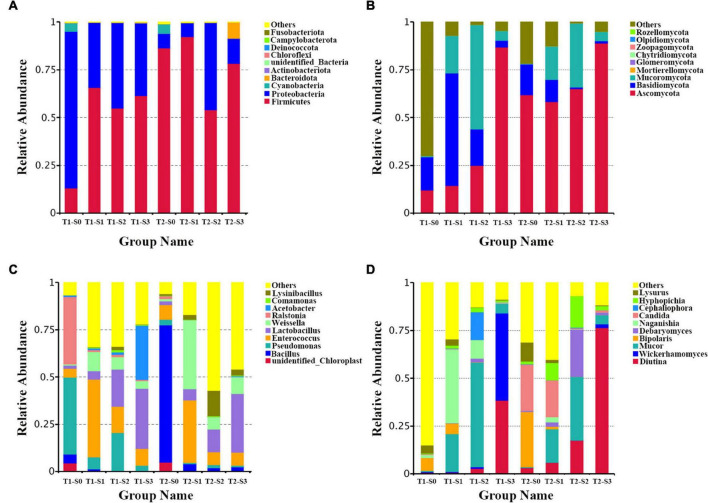
The composition of bacterial and fungal communities in fermented rapeseed cake. **(A)** The histogram of bacterial composition in fermented rapeseed cake at the phylum level. **(B)** The histogram of fungal composition in fermented rapeseed cake at the phylum level. **(C)** The histogram of bacterial composition in fermented rapeseed cake at the genus level. **(D)** The histogram of fungal composition in fermented rapeseed cake at the genus level.

In order to intuitively study the species with a higher relative abundance, the top 10 phyla and genera were selected with the highest abundance according to the results of species annotation. In the bacterial community, the dominant phyla are Firmicutes, Proteobacteria, Cyanobacteria, and Bacteroidetes (Figure 2A). The dominant genera are *Bacillus*, *Pseudomonas*, *Enterococcus*, *Lactobacillus*, *Weissella*, *Ralstonia*, *Acetobacter*, *Lysinibacillus* and *Comamonas* (Figure 2C). In the fungal community, the dominant phyla are Ascomycota, Basidiomycota, Mucoromycota, Mortierellomycota, and Glomeromycota (Figure 2B). The dominant genera are *Diotina*, *Wickerhamomyces*, *Mucor*, *Bipolaris*, *Debaryomyces*, *Naganishia*, *Candida*, *Cephaliophor*, and *Lysurus* (Figure 2D).

**FIGURE 3:**
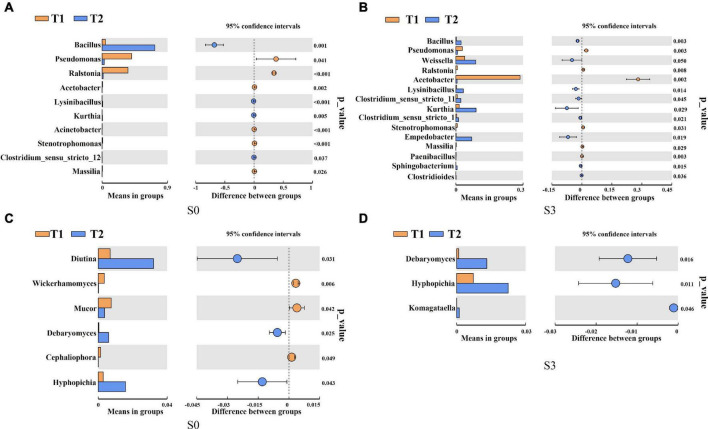
*T*-test of bacterial and fungal communities in fermented rapeseed cake. **(A)** The *t*-test of bacterial communities in fermented rapeseed cake in S0. **(B)** The *t*-test of bacterial communities in fermented rapeseed cake in S3. **(C)** The *t*-test of fungal communities in fermented rapeseed cake in S0. **(D)** The *t*-test of fungal communities in fermented rapeseed cake in S3.

To further determine the microbial community composition at the genus level of rapeseed cake under different fermentation methods, we screened the bacterial and fungal communities with significant differences in abundance by using the inter-group *t*-test. For the bacterial community, in S0, the relative abundance of *Bacillus* in T2 treatment was significantly higher than that in T1 (Figure 3A). In S3, the relative abundance of *Bacillus*, *Lysinibacillus*, and *Empedobacter* in T2 were significantly higher than those in T1 (Figure 3B). In the fungal community, in S0, *Diutina*, *Debaryomyces*, and *Hyphopichia* in T2 were significantly higher than those in T1 (Figure 3C). In S3, *Debaryomyces*, *Hyphopichia*, and *Komagataella* in T2 were significantly higher than those in T1 (Figure 3D).

### Analysis of metabolites in different fermentation methods and stages of rapeseed cake

**FIGURE 4:**
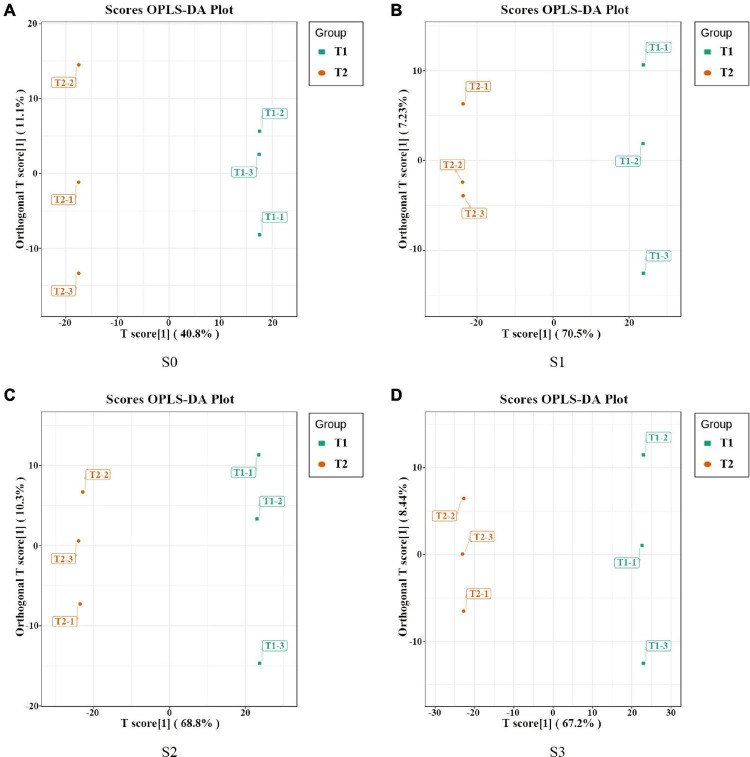
OPLS-DA analysis of metabolites in fermented rapeseed cake. **(A)** OPLS-DA analysis of metabolites in fermented rapeseed cake in S0. **(B)** OPLS-DA analysis of metabolites in fermented rapeseed cake in S1. **(C)** OPLS-DA analysis of metabolites in fermented rapeseed cake in S2. **(D)** OPLS-DA analysis of metabolites in fermented rapeseed cake in S3.

**FIGURE 5:**
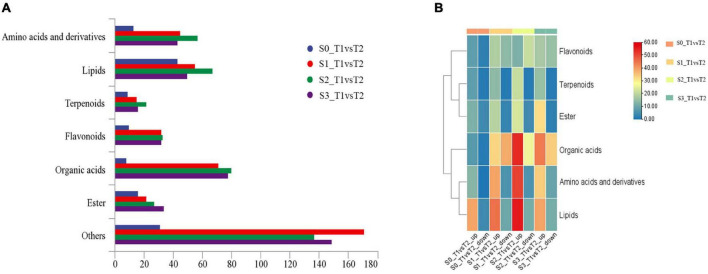
**(A)** Column chart of metabolites in fermented rapeseed cake in four stages. **(B)** Cluster heat map of metabolites in fermented rapeseed cake in four stages.

In order to further analyze the metabolites of rapeseed cake under different fermentation methods, firstly, we identified the metabolites in T1 and T2 by GC-MS and LC-MS. A total of 1,055 metabolites were detected, mainly including organic acids, amino acids and their derivatives, lipids, flavonoids, terpenoids, esters, and aromatics. Secondly, OPLS-DA analysis was performed on the metabolites in four stages to maximize the differentiation between groups (Figure 4). The results showed that T1 and T2 were well separated in different stages, indicating that the metabolites of rapeseed cake changed significantly after adding enzymatic preparation. To further compare the differences of metabolites between T1 and T2, fold_change value (≥ 2, ≤ 0.5) and VIP value (≥ 1) were used to screen the differential metabolites in four stages, and 130, 411, 423 and 402 substances were screened, respectively (Supplementary Tables 2–5). During the whole fermentation process, organic acids, amino acids and their derivatives, lipids, terpenoids, esters, and flavonoids were significantly changed after adding enzymatic preparation (Figure 5A). With continuous fermentation, amino acids and their derivatives, lipids, and organic acids were significantly increased in T2 (Figure 5B).

During the whole fermentation process, the addition of high-efficiency enzymatic preparation significantly increased the levels of some lipids, mainly including methyl linolenate, (9Z,11E)-13-oxooctadecadecadecadeca-9,11-dienoic acid, 9-hydroxy-10,12,15-octadecaterenoic acid, 1-eicosanol, ricinoleic acid, 9oxo-10E,12Z-octadecadienoic acid, arachidic acid, and crepenynic acid. While methyl palmitate and 1-linoleoylglycerol were significantly decreased. In S2 and S3, 12-hydroxystearic acid, (9Z, 12Z)–(7S, 8S)-dihydroxyadeca-9,12-dienoic acid, (12Z)-9,10-dihydroxyadec-12-enoic acid, hexadecanedioic acid were significantly increased, while 5,8,11,14-pentadecanoamide, 1-monomyristin, and 17-Hydroxylinolenic acid were significantly decreased.

During the whole fermentation process, the addition of high-efficiency enzymatic preparation significantly increased some amino acids, mainly including L-Alanyl-L-Phenylalanine, L-Alanyl-L-Phenylalanine, L-Valyl-L-Leucine, L-Glycyl-L-Isoleucine, N-Glycyl-L-Leucine, L-Phenylalanine and L-Methionine. While L-Methionine Sulfoximine was significantly decreased. In S2 and S3, Hexanoyl-l-glycine, N6-acetyl-l-lysine, L-Isoleucine, L-Norleucine, L-Leucine, N,N-Dimethylglycine, and N-Acetyl-L-Methionine were significantly increased. While L-Proline was significantly decreased.

During the whole fermentation process, the addition of high-efficiency enzymatic preparation significantly increased some organic acids, mainly including: β- Hydroxyisovaleric acid, 2-Hydroxyisohexanoic acid, argininosuccinic acid, ethyl caffeic, trigallic acid, ethyl ferulate, phenethyl caffeic, and 3-Methoxybenzoic acid. While 3,4-Dihydroxyphenylacetic acid, 3-O-Methylgallic acid, 6′-O-Sinapoylsucrose, Gentisic Acid, Salicylic acid, and 4-Hydroxy-3-Methoxybenzaldehyde were significantly decreased. In S2 and S3, 4-Methylpentanoic acid, 6-Aminocaproic acid, L-Citric acid, 5-Acetamidopentanoic acid, 2-Methylsuccinic acid, Dimethylmalonic acid, Rosmarinic acid, Sinapic acid, etc. While Citric acid, (E)-3-(3,4-dihydroxyphenyl) acrylaldehyde, 4-Hydroxybenzoic acid, 6′-O-Feruloyl-D-sucrose, 1,3-O-Diferoyloylglycerol, and Hydroxyphenyllactic acid were significantly decreased.

### Kyoto encyclopedia of genes and genomes pathway analysis of metabolites in rapeseed cake at different stages of fermentation

**FIGURE 6:**
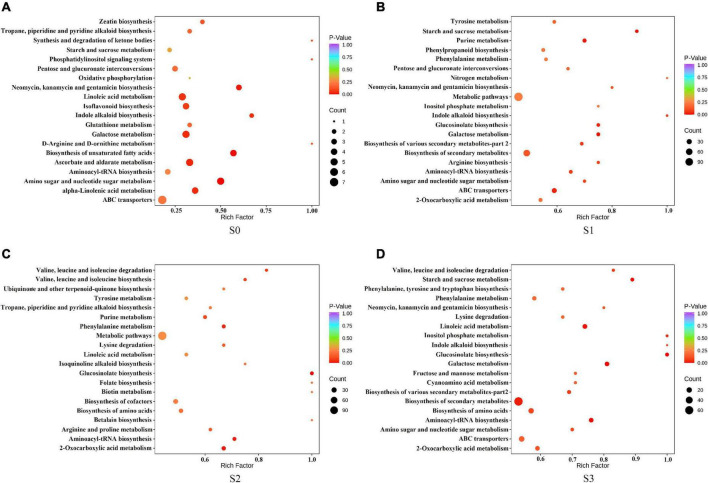
Kyoto encyclopedia of genes and genomes (KEGG) enrichment pathway of different metabolites in fermented rapeseed cake. **(A)** KEGG enrichment pathway of different metabolites in fermented rapeseed cake in S0. **(B)** KEGG enrichment pathway of different metabolites in fermented rapeseed cake in S1. **(C)** KEGG enrichment pathway of different metabolites in fermented rapeseed cake in S2. **(D)** KEGG enrichment pathway of different metabolites in fermented rapeseed cake in S3.

To further explore the role of different metabolites in different fermentation methods, we enriched the selected compounds by the KEGG pathway. The results showed that, in S0, the differential metabolites were mainly enriched in amino sugar and nucleotide sugar metabolism; biosynthesis of unsaturated fatty acids; neomycin, kanamycin, and gentamicin biosynthesis; ascorbate and aldate metabolism; alpha-Linolenic acid metabolism; galactose metabolism and indole alkaloid biosynthesis (Figure 6A). In S1, the differential metabolites were mainly enriched in starch and sucrose metabolism, galactose metabolism, purine metabolism, glucosinolate biosynthesis, and ABC transporters (Figure 6B). In S2, the differential metabolites were mainly enriched in glucosinolate biosynthesis, 2-Oxocarboxylic acid metabolism, aminoacyl-tRNA biosynthesis, and phenylalanine metabolism (Figure 6C). In S3, the differential metabolites were mainly enriched in glucosinolate biosynthesis, galactose metabolism, aminoacyl tRNA biosynthesis, starch and sucrose metabolism, linoleic acid metabolism, biosynthesis of secondary metabolites, and inositol phosphate metabolism (Figure 6D).

### Correlation analysis between enzyme activities, microbial communities, and metabolites in final products of rapeseed

**FIGURE 7:**
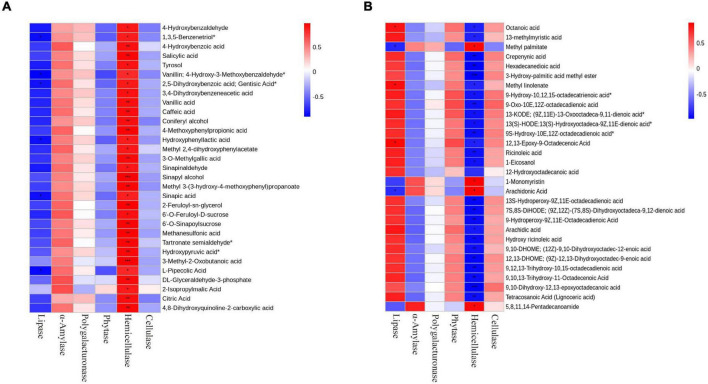
Correlation diagram of six enzymes with metabolites in fermented rapeseed cake. **(A)** Correlation diagram of six enzymes with lipids in S3. **(B)** Correlation diagram of six enzymes with Organic acids in S3.

In order to study the effect of enzymatic addition on fermentation products, we analyzed the correlation between enzymatic activity and differential metabolites. Heatmaps were prepared by using the differential metabolites and enzymatic activities with correlation (*R*^2^ ≥ 0.64, *p* < 0.05) (Figure 7). The results showed that lipase was significantly correlated with lipids, mainly including octanoic acid, methyl linolenate, and 12,13-Epoxy-9-Octadecenoic acid (Figure 7A). Hemicellulase was significantly related to organic acids, mainly including Sinapyl alcohol, 3-Methyl-2-Oxobutanoic acid, L-Cyclopentylglycine, N-Methyl-Trans-4-Hydroxy-L-Proline, γ-Glutamyltyrosine and 4-Methoxyphenylpropionic acid (Figure 7B). And α-amylase, phytase, polygalacturonase, and cellulase were not significantly correlated with metabolites.

**FIGURE 8:**
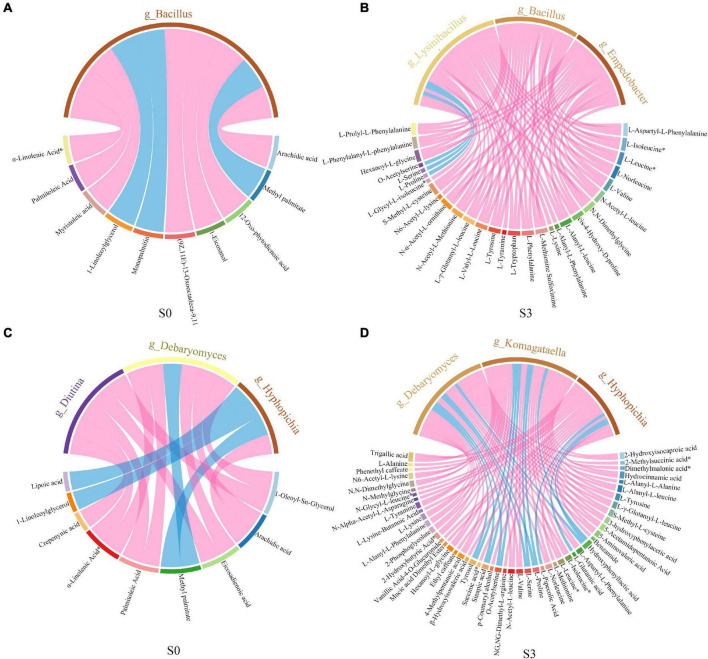
Correlation chord diagram of microorganisms and metabolites. **(A)** Correlation chord diagram of bacteria and metabolites in S0. **(B)** Correlation chord diagram of bacteria and metabolites in S3. **(C)** Correlation chord diagram of fungus and metabolites in S0. **(D)** Correlation chord diagram of fungus and metabolites in S3.

In order to study the correlation between differential microbiota and differential metabolites, spearman correlation was used to analyze the relationship between microorganisms and metabolites. The chord diagram was plotted by using the differential metabolites and microorganisms with correlation (*R*^2^ ≥ 0.64, *p* < 0.05) (Figure 8). The results showed that, in S0, the microbial community was significantly correlated with lipids by adding enzymatic preparation. *Bacillus* was significantly correlated with lipids, mainly including arachidic acid, 12-Oxo phytodienoic acid, 1-Eicosanol, 13 kode; (9Z, 11E)-13-Oxooctadeca-9,11-dienoic acid, myristoleic acid, α-Linolenic acid and palmitoleic acid (Figure 8A). *Diutina* and *Debaryomyces* were significantly correlated with 1-Oleoyl-Sn-Glycerol, palmitoleic acid, α-Linolenic acid, and eicosadienoic acid. *Debaryomyces* and *Hyphopichia* were significantly correlated with arachidic acid (Figure 8C). In S3, after adding enzymatic preparation, the microbial community was significantly correlated to amino acids and their derivatives. *Bacillus*, *Lysinibacillus* and *Empedobacter* were significantly correlated with L-valine, L-Tyrosine, L-Leucine, L-Tryptophan, L-Phenylalanine, L-Norleucine, L-Isoleucine and some derivatives (Figure 8B). *Komagataella* and *Debaryomyces* were significantly correlated to L-Tyrosine, L-Valine, L-Norleucine, L-Isoleucine, L-Leucine, L-Valine, and some derivatives. While *Hyphopichia* was significantly correlated to successful acid, L-Alanine, and some derivatives (Figure 8D).

## Discussion

### The microbial diversity induced by enzyme preparation improves the efficiency of rapeseed cake fermentation

Microbial diversity is of great significance to fermentation and promotes the flavor and stability of fermented products (21–23). According to the previous study, natural fermentation is an effective measure to maximize the role of “native microorganisms” (24). During the fermentation of Chinese liquor grains, the existence of hydrolase promotes the increase of bacterial diversity (25). In this study, it was obvious that the bacterial diversity of natural fermentation was significantly higher than that of enzymatic fermentation in the early and middle stages. While the bacterial diversity of enzymatic fermentation was significantly higher than that of natural fermentation in the late stage. We speculate that the addition of enzymatic preparation introduces a large number of *Bacillus* to participate in the fermentation process, thus inhibiting the growth of the original microbes and reducing the bacterial diversity of the rapeseed cake with enzymatic fermentation in the early and middle stages. With the progress of fermentation, the existence of hydrolase promotes the increase of bacterial diversity. The fungal diversity of enzymatic fermentation was higher than that of natural fermentation in the non-fermented and early stages. This could be because the addition of enzymatic preparation accelerated the degradation of macromolecules and increased the temperature, providing a more suitable environment for the survival of fungi. With the progress of fermentation, a suitable fermentation microenvironment was established, and the diversity of fungal species tended to be the same.

Previous studies showed that in the process of solid-state fermentation, high concentrations of volatile compounds produced by *Bacillus* and *Empedobacter* were significantly related to the volatile aroma of the fermentation products (26–28). *Bacillus* can reduce the anti-nutritional factors during soybean meal fermentation, which is conducive to improving the quality of fermented products (29). *Lysinibacillus* can degrade urea produced by solid-state fermentation and reduce toxic substances in fermentation products (30, 31). In this study, although the dominant phyla in natural fermentation and enzymatic fermentation are the same, their abundance is different. The main performance is that the abundance of Firmicutes, Bacteroidetes, and Ascomycota by enzymatic fermentation is significantly higher than that in natural fermentation. After fermentation, the relative abundance of *Bacillus*, *Lysinibacillus*, *Empedobacter*, *Debaryomyces*, *Hyphopichia*, and *Komagataella* in enzymatic fermentation was significantly higher than that in natural fermentation (Figures 3B,D). The results showed that, unlike natural fermentation, microbial diversity during enzyme fermentation is specific, which improves fermentation efficiency.

### Enzymatic fermentation promotes the conversion of macromolecular substances of rapeseed cake

Through the analysis of KEGG pathway enrichment, it was obviously found that, unlike natural fermentation, the metabolite enrichment pathway in the process of enzymatic fermentation was specific, and mostly concentrated in sugar metabolism and fatty acid metabolism. The composition of metabolites is an important indicator to judge the degree of fermentation (32–34). With the continuous fermentation of rapeseed cake, the metabolites increased gradually. More substances tended to be up-regulated after adding high-efficiency enzymatic preparation, such as fatty acids, organic acids, amino acids and their derivatives. This further shows that the addition of enzymatic preparation helps to promote the fermentation of rapeseed cake, and more organic matter is decomposed into small molecules.

Previous studies have shown that bacteria release lipase during fermentation (35). Lipid hydrolysis can explain the increase of saturated medium chain fatty acids after fermentation, such as palmitic acid, trans-13-octadecenoic acid, linolenic acid, stearic acid, etc. (36). In this study, with continuous fermentation, FFA gradually increased, including oleic acid, linoleic acid, linolenic acid, palmitic acid, and so on. The contents of fatty acids after enzymatic fermentation were much higher than that of natural fermentation. We speculate that one part of FFA comes from the decomposition of lipids by microbial hydrolases, and the other part comes from the synthesis of carbohydrates. It is proved that enzymatic fermentation is effective in improving the formation of fatty acids in rapeseed cake.

Organic acids are the main components of chemical substances and the intermediate products of the metabolic cycle produced by microbial processes (37). In this study, the beneficial organic acids were significantly up-regulated after enzymatic fermentation. We speculate that it is due to the presence of lactic acid bacteria in the fermentation process, which decomposes carbohydrates into more organic acids. In addition, organic acids provide an acidic environment for the fermentation process and a suitable environment for the survival of lactic acid bacteria.

There are large amounts of proteins in rapeseed cake, which can be transformed into amino acids and their derivatives through the action of enzymes and microorganisms. In this study, the contents of amino acids and their derivatives were significantly increased after enzymatic fermentation. On the one hand, under the action of microorganisms and enzymes, proteins or peptides are decomposed into small molecular amino acids and their derivatives; on the other hand, as the precursor of secondary metabolites, amino acids participate in the synthesis of other substances. Amino acids and derivatives participate in cellular immune response and affect plant growth. Several amino acids can be used as precursors for the synthesis of secondary metabolites (38–40). In addition, microbial proteinases released during fermentation also help to decompose complex proteins into their constituent amino acids. Therefore, enzymatic fermentation will further promote the application value of rapeseed cake.

### Enzymes and microorganisms jointly promote the transformation of macromolecules in the fermentation of rapeseed cake

Combining microbiomes and metabolomics, studying the relationship between metabolites and microbial community changes can provide a theoretical basis for the efficient fermentation of rapeseed cake (41, 42). Previous studies have shown that Firmicutes tend to enrich in high carbohydrate species, and can degrade macromolecular organic substances such as sugars and proteins (43). Bacteroidetes occupy a major advantage in many fermentation processes. It can decompose macromolecule compounds, including polysaccharides, fats, and proteins, into small organic acids, including monosaccharides, lower alcohols and amino acids (44, 45). In anaerobic fermentation, Proteobacteria consume volatile fatty acids, propionic acid, and butyric acid (46). In this study, a close relationship between microbial community and metabolites was found during the fermentation of rapeseed cake. The bacterial and fungal communities in fermentation products were strongly associated with metabolites. Spearman correlation chord diagram showed that bacteria and fungi were significantly correlated with lipids in the non-fermented stage. In the late stage of fermentation with enzymatic preparation, *Bacillus*, *Lysinibacillus*, *Empedobacter*, *Komagataella*, *Debaryomyces*, and *Hyphopichia* were significantly associated with organic acids and amino acids.

Hemicellulase and cellulase are important hydrolases, which are mainly used in the saccharification process (47). In this study, after adding enzymatic preparation, the existence of hemicellulase and lipase makes the conversion rate of lipids and acids higher, mainly including octanoic acid, methyl linolenate, 3-methyl-2-oxobutanoic acid, and sinapyl alcohol. The above results indicated that enzymes and microorganisms jointly promoted the conversion and degradation of macromolecules such as proteins, sugars, and fats in rapeseed cake.

## Conclusion

In this study, the addition of enzymatic preparation increases microbial diversity. The relative abundance of *Bacillus*, *Lysinibacillus*, *Empedobacter*, *Debaryomyces*, *Hyphopichia*, and *Komagataella* in enzymatic fermentation was significantly higher than that in natural fermentation. Unlike natural fermentation, microbial diversity during enzymatic fermentation is specific, which improves the efficiency of fermentation. Otherwise, enzymatic fermentation promotes the conversion of macromolecular substances of rapeseed cake, which increases small metabolites, such as fatty acids, organic acids, amino acids and their derivatives. The metabolite enrichment pathway is mostly concentrated in sugar metabolism and fatty acid metabolism. In conclusion, after adding enzymatic preparation, enzymes and microorganisms jointly promote the transformation of macromolecules during the fermentation of rapeseed cake, which laid a good foundation for further utilization of rapeseed cake.

## Data availability statement

The data presented in this study are deposited in the NCBI SRA repository, accession numbers: PRJNA869846 and PRJNA869857.

## Author contributions

YSo conducted an experiment, analyzed the data, and wrote the manuscript. SZ and YSh collected the samples. ZD, YW, and KF put forward hypotheses and designed the experiments. LS and HW reviewed the manuscript. YZS, WW, JZ, YM, and XH participated in the experimental design and guided the research. All authors contributed to the article and approved the submission.

## Footnotes

1
http://ccb.jhu.edu/software/FLASH/

2
http://qiime.org/scripts/split_libraries_fastq.html

3
https://github.com/torognes/vsearch/

4
http://www.drive5.com/uparse/

5
http://www.arb-silva.de/

6
http://unite.ut.ee/

7
http://qiime.org/scripts/assign_taxonomy.html

8
http://www.drive5.com/muscle/

9
http://www.kegg.jp/kegg/compound/

10
http://www.kegg.jp/kegg/pathway.html
