# Supplementary material for: Enzymes and microorganisms jointly promote the fermentation of rapeseed cake
Source: Front Nutr. 2022 Sep 15;9:989410. doi: 10.3389/fnut.2022.989410 (PMC9521174; doi:10.3389/fnut.2022.989410)
Supplement: Supplementary file 1 [file Data_Sheet_1.docx]

| **Stage** | **Samples** | | **Zn mg/kg** | **Mg g/kg** | **Cu mg/kg** | **Mn mg/kg** | **Ca g/kg** | **Fe g/kg** |
| --- | --- | --- | --- | --- | --- | --- | --- | --- |
| S0 | | T1 | 61.27±1.82a | 10.91±1.18a | 1.01±0.17a | 67.17±9.85a | 1.79±0.13a | 0.15±0.02a |
|  |  | T2 | 62.01±4.73a | 10.64±0.44a | 0.55±0.26b | 60.83±6.29b | 1.79±0.04a | 0.14±0.01a |
| S1 | | T1 | 77.57±0.96a | 10.59±0.15b | 1.42±0.48b | 59.13±3.26b | 2.060.15b | 0.20±0.03a |
|  |  | T2 | 72.76±3.57b | 11.23±0.44a | 1.97±0.99a | 90.36±3.69a | 2.36±0.17a | 0.16±0.03b |
| S2 | | T1 | 83.09±6.50a | 11.01±0.34a | 1.57±0.67b | 90.97±6.12a | 2.42±0.13a | 0.23±0.04a |
|  |  | T2 | 77.68±4.14b | 11.27±1.25a | 1.76±0.42a | 93.62±9.26a | 2.43±0.02a | 0.19±0.04b |
| S3 | | T1 | 87.72±2.71a | 11.32±0.46a | 1.94±0.25a | 93.88±4.73a | 2.58±0.10a | 0.20±0.02a |
|  |  | T2 | 85.54±1.85a | 11.91±0.56a | 1.94±0.32a | 95.82±1.02a | 2.61±0.12a | 0.18±0.01a |

Table S1 The contents of mineral elements in T1 and T2 in fermented rapeseed cake in four stages

Table S2 Differential metabolites of T1 vs T2 in S0

| **Class** | **Compounds** | **VIP** | **Fold_Change** | **Type** |
| --- | --- | --- | --- | --- |
| Amino acids and their derivatives | L-Alanyl-L-Phenylalanine | 1.57 | 36003.70 | up |
|  | L-Aspartyl-L-Phenylalanine | 1.56 | 4534.56 | up |
|  | L-Histidine | 1.11 | 2178.33 | up |
|  | L-Alanyl-L-Alanine | 1.57 | 1759.67 | up |
|  | γ-Glutamylphenylalanine | 1.11 | 1264.89 | up |
|  | L-Leucyl-L-phenylalanine | 1.11 | 1167.70 | up |
|  | L-Ornithine | 1.57 | 540.37 | up |
|  | L-Valyl-L-Leucine | 1.56 | 18.74 | up |
|  | L-Glycyl-L-isoleucine* | 1.54 | 15.09 | up |
|  | N-Glycyl-L-leucine* | 1.55 | 12.64 | up |
|  | L-Phenylalanine | 1.54 | 5.07 | up |
|  | L-Methionine | 1.49 | 3.26 | up |
|  | L-Methionine Sulfoximine | 1.03 | 2.13 | up |
| Lipids | LysoPC 19:2(2n isomer) | 1.57 | 20192.96 | up |
|  | 2-α-Linolenoyl-glycerol-1,3-di-O-glucoside | 1.57 | 11048.59 | up |
|  | LysoPE 15:0(2n isomer) | 1.56 | 1785.96 | up |
|  | Myristoleic acid | 1.57 | 1527.26 | up |
|  | 1-α-Linolenoyl-glycerol | 1.57 | 133.05 | up |
|  | 2-α-Linolenoyl-glycerol | 1.57 | 125.70 | up |
|  | 1-Stearidonoyl-Glycerol | 1.35 | 86.68 | up |
|  | Methyl linolenate | 1.56 | 67.80 | up |
|  | 2-Linoleoylglycerol-1,3-di-O-glucoside | 1.56 | 48.80 | up |
|  | 1-Oleoyl-Sn-Glycerol | 1.56 | 45.60 | up |
|  | 2-Linoleoylglycerol | 1.56 | 44.44 | up |
|  | 13-KODE;(9Z,11E)-13-Oxooctadeca-9,11-dienoic acid* | 1.56 | 13.08 | up |
|  | 9-Hydroxy-10,12,15-octadecatrienoic acid* | 1.56 | 12.90 | up |
|  | 1-Eicosanol | 1.56 | 10.44 | up |
|  | Ricinoleic acid | 1.56 | 10.22 | up |
|  | 9-Oxo-10E,12Z-octadecadienoic acid | 1.56 | 9.56 | up |
|  | 9-Hydroxy-12-oxo-10(E),15(Z)-octadecadienoic acid | 1.55 | 9.02 | up |
|  | LysoPE 16:1(2n isomer) | 1.55 | 7.54 | up |
|  | Arachidic acid | 1.55 | 5.16 | up |
|  | LysoPC 19:1 | 1.28 | 5.13 | up |
|  | LysoPE 16:3 | 1.47 | 4.77 | up |
|  | LysoPC 16:1(2n isomer) | 1.56 | 4.23 | up |
|  | LysoPI 16:0 | 1.55 | 3.28 | up |
|  | Eicosadienoic acid | 1.56 | 3.08 | up |
|  | LysoPC 20:2(2n isomer) | 1.54 | 2.94 | up |
|  | LysoPC 17:2 | 1.52 | 2.90 | up |
|  | LysoPE 17:1 | 1.53 | 2.87 | up |
|  | 12,13-Epoxy-9-Octadecenoic Acid | 1.54 | 2.71 | up |
|  | LysoPE 18:2(2n isomer) | 1.51 | 2.58 | up |
|  | LysoPE 18:3(2n isomer) | 1.48 | 2.55 | up |
|  | LysoPC 20:1 | 1.51 | 2.50 | up |
|  | LysoPE 18:1(2n isomer) | 1.51 | 2.29 | up |
|  | 12-Oxo-phytodienoic acid | 1.30 | 2.17 | up |
|  | LysoPC 18:1 | 1.55 | 2.14 | up |
|  | Palmitoleic Acid | 1.44 | 2.14 | up |
|  | Crepenynic acid | 1.55 | 2.11 | up |
|  | α-Linolenic Acid* | 1.55 | 2.09 | up |
|  | γ-Linolenic Acid* | 1.55 | 2.07 | up |
|  | LysoPC 18:2 | 1.55 | 2.07 | up |
|  | Methyl palmitate | 1.42 | 0.46 | down |
|  | Monopalmitin | 1.53 | 0.28 | down |
|  | Pentadecanoic Acid | 1.50 | 0.24 | down |
|  | 1-Linoleoylglycerol | 1.57 | 0.00 | down |
| Terpenoids | Oleanolic acid | 1.57 | 6895.33 | up |
|  | Soyasaponin βc | 1.57 | 1620.78 | up |
|  | Soyasapogenol B-3-O-glucuronide | 1.56 | 724.20 | up |
|  | Soyasaponin IV | 1.56 | 651.13 | up |
|  | Soyasaponin βe | 1.56 | 520.44 | up |
|  | SoyasapogenolE-3-Orhamnosyl(1,2)glucosyl(1,2)glucuronide | 1.11 | 485.37 | up |
|  | 24,30-Dihydroxy-12(13)-enolupinol | 1.56 | 113.65 | up |
|  | Betulinic acid | 1.53 | 8.36 | up |
|  | 3-Cyclohexen-1-ol, 4-methyl-1-(1-methylethyl)-, (R)- | 1.16 | 0.35 | down |
| Flavonoids | Daidzein-7-O-glucoside(Daidzin) | 1.56 | 4540.37 | up |
|  | 3',7-dihydroxy-4'-methoxyflavone | 1.56 | 3034.22 | up |
|  | Glycitin | 1.11 | 1459.00 | up |
|  | Genistein | 1.11 | 1117.30 | up |
|  | Apigenin-7-O-(6''-acetyl)glucoside | 1.11 | 1002.11 | up |
|  | 6''-O-Acetylgenistin | 1.11 | 987.15 | up |
|  | Daidzein | 1.22 | 10.22 | up |
|  | Quercetin | 1.10 | 2.90 | up |
|  | Apigenin-8-C-Glucoside (Vitexin) | 1.12 | 0.49 | down |
|  | Aromadendrin-7-O-glucoside | 1.14 | 0.24 | down |
| Ester | Butanoic acid, propyl ester | 1.56 | 506.51 | up |
|  | Tetradecanoic acid, ethyl ester | 1.56 | 392.97 | up |
|  | 2-Methoxy-5-methylphenol, acetate | 1.56 | 343.37 | up |
|  | Isopropyl palmitate | 1.55 | 271.25 | up |
|  | Pentadecanoic acid, ethyl ester | 1.56 | 138.04 | up |
|  | trans,trans-9,12-Octadecadienoic acid, propyl ester | 1.30 | 22.60 | up |
|  | Hexadecanoic acid, ethyl ester | 1.50 | 19.42 | up |
|  | Ethyl Oleate | 1.13 | 11.28 | up |
|  | 9-Octadecenoic acid (Z)-, methyl ester | 1.40 | 9.30 | up |
|  | Octadecanoic acid, ethyl ester | 1.17 | 8.69 | up |
|  | 9,12-Octadecadienoic acid (Z,Z)-, methyl ester | 1.08 | 8.68 | up |
|  | Methyl stearate | 1.33 | 2.81 | up |
|  | Pentanoic acid, 4-methyl-, ethyl ester | 1.10 | 0.36 | down |
|  | 3-Methylbut-2-enoic acid, 2-methylpentyl ester | 1.12 | 0.29 | down |
|  | 2-(2-butoxyethoxy)-Ethanol,acetate | 1.52 | 0.22 | down |
|  | Hexadecanoic acid, butyl ester | 1.54 | 0.01 | down |
| Organic acids | 2,6-Dimethoxybenzaldehyde* | 1.39 | 2.83 | up |
|  | 2-Hydroxy-3-phenylpropanoic acid | 1.54 | 2.91 | up |
|  | 3-(4-Hydroxyphenyl)-propionic acid* | 1.55 | 2.73 | up |
|  | 1-O-p-Hydroxycinnamoyl-3-O-caffeoylglycerol | 1.34 | 2.64 | up |
|  | Butanoic Acid, 2-methyl- | 1.10 | 692.12 | up |
|  | 3-Hydroxybutyric acid | 1.57 | 11167.89 | up |
|  | N-Methyl-4-aminobutyric acid | 1.20 | 6.17 | up |
|  | Lipoic acid | 1.56 | 0.00 | down |
| Others | N-(beta-D-Glucosyl)nicotinate | 1.56 | 31451.85 | up |
|  | 1,6-anhydro-β-D-glucose | 1.48 | 6.84 | up |
|  | D-Mannose* | 1.49 | 4.94 | up |
|  | D-Glucose* | 1.42 | 4.48 | up |
|  | Inositol* | 1.47 | 4.20 | up |
|  | D-Fructose* | 1.47 | 4.13 | up |
|  | D-Galactose* | 1.49 | 4.11 | up |
|  | D-Galacturonic acid* | 1.49 | 2.66 | up |
|  | D-Arabinose | 1.41 | 2.10 | up |
|  | L-Ascorbic acid (Vitamin C) | 1.11 | 0.34 | down |
|  | Tryptamine | 1.57 | 3867.81 | up |
|  | N-Acetylputrescine | 1.57 | 2717.11 | up |
|  | N,N'-Diferuloylputrescine | 1.17 | 5.82 | up |
|  | N-Benzylmethylene isomethylamine | 1.55 | 5.63 | up |
|  | Acetryptine | 1.54 | 3.20 | up |
|  | Uridine 5'-diphospho-D-glucose | 1.55 | 3509.54 | up |
|  | Nicotinic acid adenine dinucleotide | 1.54 | 2451.10 | up |
|  | Uridine 5'-diphospho-N-acetylglucosamine | 1.57 | 586.36 | up |
|  | 6-Methylmercaptopurine | 1.55 | 5.82 | up |
|  | β-Pseudouridine | 1.15 | 5.15 | up |
|  | Uridine 5'-diphosphate | 1.45 | 3.82 | up |
|  | Isopentenyladenine-7-N-glucoside | 1.18 | 2.16 | up |
|  | 1-Methylxanthine | 1.11 | 0.01 | down |
|  | Berteroin | 1.56 | 587.30 | up |
|  | Sulfamide | 1.50 | 0.25 | down |
|  | 2-n-Propylaziridine | 1.10 | 841.53 | up |
|  | 7,9-Di-tert-butyl-1-oxaspiro(4,5)deca-6,9-diene-2,8-dione | 1.21 | 2.41 | up |
|  | 1-Pentanol | 1.10 | 1663.49 | up |
|  | 3-Octanol | 1.56 | 174.95 | up |
|  | 1-Hexadecanol | 1.36 | 0.49 | down |
|  | Oxirane, tetradecyl- | 1.35 | 2.04 | up |

* denote substances with isomers that are difficult to distinguish by mass spectrometry

Table S3 Differential metabolites of T1 vs T2 in S1

| **Class** | **Compounds** | **VIP** | **Fold_Change** | **Type** | |
| --- | --- | --- | --- | --- | --- |
| Amino acids and their derivatives | L-Aspartyl-L-Phenylalanine | 1.19 | 91928.15 | up |  |
|  | L-Tyramine | 1.19 | 12710.67 | up |  |
|  | Phenylacetyl-L-glutamine | 1.19 | 4249.41 | up |  |
|  | Tryptophan glutamic acid | 1.19 | 2702.85 | up |  |
|  | N-Methylglycine | 1.19 | 2163.15 | up |  |
|  | L-Valyl-L-Leucine | 1.12 | 88.47 | up |  |
|  | L-Glycyl-L-isoleucine* | 1.13 | 86.35 | up |  |
|  | N-Glycyl-L-leucine* | 1.12 | 84.24 | up |  |
|  | L-Phenylalanyl-L-phenylalanine | 1.01 | 73.94 | up |  |
|  | L-Alanyl-L-Phenylalanine | 1.16 | 68.34 | up |  |
|  | L-Phenylalanine | 1.15 | 54.42 | up |  |
|  | L-Methionine | 1.15 | 49.90 | up |  |
|  | L-Valine | 1.17 | 41.09 | up |  |
|  | L-Tyrosine | 1.15 | 37.93 | up |  |
|  | L-Lysine | 1.18 | 33.04 | up |  |
|  | L-Glutamine | 1.18 | 27.68 | up |  |
|  | Glycyl-tryptophan | 1.18 | 24.04 | up |  |
|  | L-Prolyl-L-Phenylalanine | 1.16 | 22.21 | up |  |
|  | S-Methyl-L-cysteine | 1.18 | 19.67 | up |  |
|  | L-Alanyl-L-Alanine | 1.17 | 15.44 | up |  |
|  | L-Ornithine | 1.17 | 9.80 | up |  |
|  | L-threo-3-Methylaspartate | 1.18 | 9.80 | up |  |
|  | L-Tryptophan | 1.17 | 7.55 | up |  |
|  | NG,NG-Dimethyl-L-arginine | 1.18 | 7.31 | up |  |
|  | L-Histidine | 1.18 | 6.95 | up |  |
|  | -glutamylmethionine | 1.19 | 5.44 | up |  |
|  | N--Acetyl-L-ornithine | 1.10 | 4.66 | up |  |
|  | N-Alpha-Acetyl-L-Asparagine | 1.15 | 4.56 | up |  |
|  | L-Lysine-Butanoic Acid | 1.11 | 4.52 | up |  |
|  | L--Glutamyl-L-leucine | 1.16 | 4.13 | up |  |
|  | L-Glutamic acid | 1.17 | 3.69 | up |  |
|  | L-Azetidine-2-carboxylic acid | 1.18 | 3.55 | up |  |
|  | L-Serine | 1.17 | 2.96 | up |  |
|  | N-Acetyl-L-threonine | 1.09 | 2.91 | up |  |
|  | Trimethyllysine | 1.14 | 2.87 | up |  |
|  | L-Alanyl-L-leucine | 1.15 | 2.50 | up |  |
|  | L-Methionine Sulfoximine | 1.11 | 2.48 | up |  |
|  | cis-4-Hydroxy-D-proline | 1.15 | 2.33 | up |  |
|  | N-Acetyl-L-leucine | 1.14 | 2.01 | up |  |
|  | N-acetyl-beta-alanine | 1.15 | 0.50 | down |  |
|  | N-Acetyl-L-glutamic acid | 1.17 | 0.43 | down |  |
|  | S-(Methyl)glutathione | 1.19 | 0.24 | down |  |
|  | N-Acetyl-L-Tryptophan | 1.09 | 0.18 | down |  |
|  | N-Methyl-Trans-4-Hydroxy-L-Proline | 1.17 | 0.08 | down |  |
|  | -Glutamylphenylalanine | 1.19 | 0.00 | down |  |
| Lipids | LysoPC 20:3 | 1.19 | 16490.00 | up |  |
|  | Octanoic acid | 1.19 | 515.56 | up |  |
|  | Methyl linolenate | 1.12 | 27.26 | up |  |
|  | 9-Oxo-10E,12Z-octadecadienoic acid | 1.17 | 13.38 | up |  |
|  | LysoPE 16:1 | 1.19 | 12.82 | up |  |
|  | 9-Hydroxy-10,12,15-octadecatrienoic acid* | 1.17 | 11.45 | up |  |
|  | 13-KODE; (9Z,11E)-13-Oxooctadeca-9,11-dienoic acid* | 1.17 | 11.39 | up |  |
|  | LysoPC 17:2 | 1.18 | 9.36 | up |  |
|  | Ricinoleic acid | 1.16 | 9.24 | up |  |
|  | 1-Eicosanol | 1.17 | 9.21 | up |  |
|  | 2-Linoleoylglycerol-1,3-di-O-glucoside | 1.03 | 7.31 | up |  |
|  | 2--Linolenoyl-glycerol | 1.04 | 7.13 | up |  |
|  | LysoPC 16:1(2n isomer) | 1.18 | 7.12 | up |  |
|  | 1--Linolenoyl-glycerol | 1.03 | 6.94 | up |  |
|  | LysoPE 18:3(2n isomer) | 1.18 | 6.41 | up |  |
|  | 2-Linoleoylglycerol | 1.05 | 6.12 | up |  |
|  | LysoPE 16:3 | 1.16 | 5.18 | up |  |
|  | LysoPE 16:1(2n isomer) | 1.13 | 5.13 | up |  |
|  | LysoPI 16:0 | 1.18 | 5.10 | up |  |
|  | LysoPE 18:2(2n isomer) | 1.18 | 4.33 | up |  |
|  | 9-Oxo-12Z-Octadecenoic acid* | 1.17 | 4.21 | up |  |
|  | 12-Oxo-phytodienoic acid | 1.15 | 3.76 | up |  |
|  | 12,13-Epoxy-9-Octadecenoic Acid | 1.18 | 3.76 | up |  |
|  | Myristoleic acid | 1.17 | 3.64 | up |  |
|  | LysoPC 18:2 | 1.17 | 3.41 | up |  |
|  | LysoPC 19:1 | 1.14 | 3.36 | up |  |
|  | 13S-Hydroperoxy-9Z,11E-octadecadienoic acid | 1.18 | 3.30 | up |  |
|  | 9-Hydroperoxy-10E,12,15Z-octadecatrienoic acid | 1.18 | 3.30 | up |  |
|  | Tetracosanoic Acid (Lignoceric acid) | 1.13 | 3.29 | up |  |
|  | 9,10-Epoxyoctadecanoic Acid | 1.16 | 3.26 | up |  |
|  | LysoPC 19:2 | 1.12 | 3.10 | up |  |
|  | LysoPE 18:1(2n isomer) | 1.17 | 3.10 | up |  |
|  | LysoPE 18:3 | 1.16 | 3.02 | up |  |
|  | LysoPC 19:2(2n isomer) | 1.08 | 2.72 | up |  |
|  | Arachidic acid | 1.17 | 2.71 | up |  |
|  | LysoPE 15:0(2n isomer) | 1.14 | 2.67 | up |  |
|  | 13(S)-HODE;13(S)-Hydroxyoctadeca-9Z,11E-dienoic acid* | 1.18 | 2.60 | up |  |
|  | LysoPC 18:1 | 1.17 | 2.56 | up |  |
|  | 3-Hydroxy-palmitic acid methyl ester | 1.17 | 2.42 | up |  |
|  | 10-Heptadecenoic Acid | 1.18 | 2.36 | up |  |
|  | 9S-Hydroxy-10E,12Z-octadecadienoic acid* | 1.18 | 2.35 | up |  |
|  | Hydroxy ricinoleic acid | 1.16 | 2.34 | up |  |
|  | Crepenynic acid | 1.15 | 2.06 | up |  |
|  | Choline Alfoscerate | 1.07 | 2.02 | up |  |
|  | -Linolenic Acid* | 1.17 | 2.01 | up |  |
|  | LysoPC 16:2 | 1.16 | 0.48 | down |  |
|  | Methyl palmitate | 1.15 | 0.43 | down |  |
|  | Monopalmitin | 1.17 | 0.37 | down |  |
|  | Pentadecanoic Acid | 1.16 | 0.33 | down |  |
|  | LysoPC 20:2 | 1.18 | 0.27 | down |  |
|  | LysoPE 17:1 | 1.11 | 0.26 | down |  |
|  | LysoPC 20:0 | 1.18 | 0.17 | down |  |
|  | Arachidonic Acid | 1.19 | 0.00 | down |  |
|  | 1-Linoleoylglycerol | 1.19 | 0.00 | down |  |
|  | 15(R)-Hydroxylinoleic Acid* | 1.19 | 0.00 | down |  |
| Terpenoids | Cryptomeridiol | 1.19 | 17204.81 | up |  |
|  | Soyasaponin c | 1.19 | 6846.56 | up |  |
|  | Soyasaponin e | 1.19 | 2668.26 | up |  |
|  | Soyasapogenol B-3-O-glucuronide | 1.19 | 2441.11 | up |  |
|  | Soyasaponin IV | 1.19 | 2016.04 | up |  |
|  | Soyasapogenol E-3-O-rhamnosyl(1,2)glucosyl(1,2)glucuronide | 1.19 | 1751.52 | up |  |
|  | Soyasaponin a (Soyasaponin V) | 1.19 | 1124.93 | up |  |
|  | Soyasaponin III | 1.16 | 13.26 | up |  |
|  | Soyasaponin b' | 1.17 | 11.17 | up |  |
|  | 24,30-Dihydroxy-12(13)-enolupinol | 1.08 | 8.70 | up |  |
|  | Soyasaponin b (Soyasaponin I) | 1.17 | 7.92 | up |  |
|  | Betulinic acid | 1.17 | 5.13 | up |  |
|  | Betulonic acid | 1.15 | 3.28 | up |  |
|  | trans-.beta.-Ionone | 1.18 | 2.15 | up |  |
|  | Soyasapogenol B | 1.13 | 0.46 | down |  |
| Flavonoids | 2'-Hydroxygenistein | 1.19 | 9049.07 | up |  |
|  | Luteolin (5,7,3',4'-Tetrahydroxyflavone) | 1.19 | 7189.44 | up |  |
|  | 3',4',7-Trihydroxyflavone | 1.19 | 6964.96 | up |  |
|  | Calycosin | 1.19 | 4160.04 | up |  |
|  | Isoluteolin (Orobol)(5,7,3',4'-tetrahydroxyisoflavone) | 1.19 | 3359.96 | up |  |
|  | Luteolin-8-C-glucoside (Orientin) | 1.19 | 2766.00 | up |  |
|  | Glycitein | 1.19 | 2582.26 | up |  |
|  | Quercetin-7-O-rutinoside | 1.06 | 8.56 | up |  |
|  | Daidzein | 1.14 | 7.80 | up |  |
|  | Quercetin-3-O-(6''-acetyl)galactoside | 1.18 | 7.25 | up |  |
|  | Quercetin-3-O-neohesperidoside | 1.00 | 6.50 | up |  |
|  | Apigenin | 1.14 | 5.88 | up |  |
|  | Kaempferol-3-O-(6''-Sinapyl)glucosyl-(12)-Galactoside | 1.19 | 4.22 | up |  |
|  | Kaempferol-3-O-(2''-sinapoyl)glucosyl-(12)-(6''-acetyl)glucoside | 1.15 | 3.62 | up |  |
|  | Luteolin-3'-O-glucoside | 1.15 | 3.23 | up |  |
|  | Quercetin-3-O-glucoside (Isoquercitrin)* | 1.16 | 2.37 | up |  |
|  | Naringenin (5,7,4'-Trihydroxyflavanone) | 1.16 | 2.26 | up |  |
|  | Quercetin-3-O-galactoside (Hyperin) | 1.18 | 2.17 | up |  |
|  | Kaempferol (3,5,7,4'-Tetrahydroxyflavone) | 1.19 | 0.37 | down |  |
|  | Kaempferol-3-O-(2''-sinapoyl)glucosyl-(12)-(6''-malonyl)glucoside | 1.19 | 0.35 | down |  |
|  | Kaempferol-3-O-galactoside (Trifolin) | 1.19 | 0.30 | down |  |
|  | Tricin-7-O-saccharic acid | 1.19 | 0.29 | down |  |
|  | Diosmetin-7-O-rutinoside (Diosmin) | 1.17 | 0.21 | down |  |
|  | Hispidulin-7-O-(6''-O-p-Coumaroyl)Glucoside | 1.17 | 0.21 | down |  |
|  | Luteolin-7-O-(2'''-O-rhamnosyl)sophoroside-5-O-glucoside | 1.18 | 0.14 | down |  |
|  | Kaempferol-3-O-sophoroside | 1.03 | 0.01 | down |  |
|  | Kaempferol-3-O-sophorotrioside | 1.19 | 0.00 | down |  |
|  | Epicatechin glucoside | 1.07 | 0.00 | down |  |
|  | Aromadendrin (Dihydrokaempferol) | 1.19 | 0.00 | down |  |
|  | Aromadendrin-7-O-glucoside | 1.19 | 0.00 | down |  |
|  | Naringenin-7-O-glucoside (Prunin) | 1.19 | 0.00 | down |  |
|  | Tricin-7-O-(2''-O-glucosyl)glucoside | 1.19 | 0.00 | down |  |
| Ester | 2(3H)-Furanone, dihydro-5-(2-octenyl)-, (Z)- | 1.19 | 11636.50 | up |  |
|  | (Z)-Ethyl heptadec-9-enoate | 1.19 | 3986.33 | up |  |
|  | Hexadecanoic acid, propyl ester | 1.19 | 2063.97 | up |  |
|  | Benzenepropanoic acid, ethyl ester | 1.19 | 1740.63 | up |  |
|  | Butanoic acid, propyl ester | 1.19 | 850.51 | up |  |
|  | N-(3-Iodopropyl)carbamic acid, benzyl ester | 1.19 | 479.63 | up |  |
|  | 2-Methoxy-5-methylphenol, acetate | 1.19 | 479.32 | up |  |
|  | Octadecanoic acid, ethyl ester | 1.15 | 142.47 | up |  |
|  | Pentadecanoic acid, ethyl ester | 1.03 | 134.35 | up |  |
|  | Hexadecanoic acid, ethyl ester | 1.14 | 85.73 | up |  |
|  | Isopropyl palmitate | 1.07 | 58.85 | up |  |
|  | trans,trans-9,12-Octadecadienoic acid, propyl ester | 1.13 | 44.22 | up |  |
|  | Undecanoic acid, ethyl ester | 1.18 | 40.14 | up |  |
|  | Ethyl Oleate | 1.00 | 13.12 | up |  |
|  | 9,12-Octadecadienoic acid (Z,Z)-, methyl ester | 1.16 | 11.30 | up |  |
|  | Hexadecanoic acid, butyl ester | 1.18 | 10.42 | up |  |
|  | 9-Octadecenoic acid (Z)-, methyl ester | 1.15 | 9.11 | up |  |
|  | Methyl stearate | 1.14 | 3.60 | up |  |
|  | Methyl tetradecanoate | 1.14 | 3.20 | up |  |
|  | Phenethyl isocyanate | 1.11 | 0.49 | down |  |
|  | 2-(2-butoxyethoxy)-Ethanol,acetate | 1.19 | 0.06 | down |  |
|  | Pentanoic acid, 4-methyl-, ethyl ester | 1.19 | 0.00 | down |  |
| Organic acids | Ethyl caffeate | 1.19 | 17630.00 | up |  |
|  | Trigallic acid | 1.19 | 14712.96 | up |  |
|  | 4-Hydroxyphenylacetic acid | 1.19 | 3932.67 | up |  |
|  | Methyl 2,4-dihydroxyphenylacetate | 1.19 | 9.22 | up |  |
|  | Ethyl ferulate | 1.19 | 8.24 | up |  |
|  | p-Coumaryl alcohol | 1.19 | 5.51 | up |  |
|  | Phenethyl caffeate | 1.18 | 4.48 | up |  |
|  | 3,6'-Disinapoylsucrose | 1.19 | 4.30 | up |  |
|  | Caffeic acid | 1.19 | 2.80 | up |  |
|  | 3-Methoxybenzoic acid | 1.13 | 2.55 | up |  |
|  | Methyl sinapate | 1.19 | 2.51 | up |  |
|  | Ethylparaben | 1.18 | 2.51 | up |  |
|  | 1,3-O-Di-p-Coumaroylglycerol | 1.09 | 2.46 | up |  |
|  | Coniferyl alcohol | 1.19 | 2.16 | up |  |
|  | Ferulic acid* | 1.08 | 2.14 | up |  |
|  | 3-[(1-Carboxyvinyl)oxy]benzoic acid | 1.17 | 2.12 | up |  |
|  | Sinapyl alcohol | 1.17 | 2.10 | up |  |
|  | Methyl 3-(3-hydroxy-4-methoxyphenyl)propanoate | 1.07 | 2.09 | up |  |
|  | 4-Methoxyphenylpropionic acid | 1.13 | 2.08 | up |  |
|  | Homogentisic acid | 1.19 | 0.46 | down |  |
|  | 3,4-Dihydroxybenzeneacetic acid | 1.19 | 0.42 | down |  |
|  | Hydrocinnamic acid | 1.18 | 0.38 | down |  |
|  | 3-O-Methylgallic acid | 1.17 | 0.36 | down |  |
|  | 6'-O-Sinapoylsucrose | 1.19 | 0.34 | down |  |
|  | 1-O-Glucosyl sinapate | 1.18 | 0.27 | down |  |
|  | 2,5-Dihydroxybenzoic acid; Gentisic Acid* | 1.19 | 0.27 | down |  |
|  | Sinapinaldehyde | 1.19 | 0.24 | down |  |
|  | Tyrosol | 1.19 | 0.20 | down |  |
|  | Salicylic acid | 1.19 | 0.16 | down |  |
|  | 4-Hydroxybenzaldehyde | 1.17 | 0.14 | down |  |
|  | Syringaldehyde; 4-Hydroxy-3,5-Dimethoxybenzaldehyde | 1.19 | 0.11 | down |  |
|  | Isovanillin | 1.19 | 0.10 | down |  |
|  | Vanillin; 4-Hydroxy-3-Methoxybenzaldehyde* | 1.18 | 0.08 | down |  |
|  | Protocatechuic acid-4-O-glucoside* | 1.19 | 0.03 | down |  |
|  | 1-O-Gentisoyl--D-glucoside* | 1.19 | 0.02 | down |  |
|  | Dihydrocaffeoylglucose | 1.19 | 0.00 | down |  |
|  | 2-(3,4-dihydroxyphenyl)ethanediol 1-O--D-glucopyranoside | 1.19 | 0.00 | down |  |
|  | 4-O-Glucosyl-4-hydroxybenzoic acid | 1.19 | 0.00 | down |  |
|  | 1-O-Salicyloyl--D-glucose | 1.19 | 0.00 | down |  |
|  | Androsin | 1.19 | 0.00 | down |  |
|  | Demethyl coniferin | 1.19 | 0.00 | down |  |
|  | 1-O-Caffeoyl--D-glucose | 1.19 | 0.00 | down |  |
|  | Vanillic Acid-4-O-Glucuronide | 1.19 | 0.00 | down |  |
|  | 1-O-Vanilloyl-D-Glucose | 1.19 | 0.00 | down |  |
|  | Syringin | 1.19 | 0.00 | down |  |
|  | Coniferin | 1.19 | 0.00 | down |  |
|  | 4-O-Glucosyl-sinapate | 1.19 | 0.00 | down |  |
|  | 1,3,5-Benzenetriol* | 1.19 | 0.00 | down |  |
|  | 1-O-Sinapoyl-D-glucose | 1.19 | 0.00 | down |  |
|  | Vanilloloside | 1.19 | 0.00 | down |  |
|  | D-Erythronolactone | 1.19 | 3129.67 | up |  |
|  | 2-Hydroxyethylphosphonic acid | 1.19 | 19.71 | up |  |
|  | -Hydroxyisovaleric acid | 1.17 | 3.63 | up |  |
|  | 1-Aminocyclopropane-1-carboxylic acid | 1.19 | 3.44 | up |  |
|  | 2-Hydroxyisocaproic acid | 1.18 | 3.23 | up |  |
|  | Decanoic acid | 1.16 | 2.97 | up |  |
|  | DL-Glyceraldehyde-3-phosphate | 1.17 | 2.95 | up |  |
|  | Succinic acid* | 1.16 | 2.93 | up |  |
|  | Aminomalonic acid | 1.15 | 2.92 | up |  |
|  | Methylmalonic acid* | 1.16 | 2.86 | up |  |
|  | Succinic anhydride | 1.17 | 2.32 | up |  |
|  | Argininosuccinic acid | 1.17 | 2.25 | up |  |
|  | 5-Aminovaleric acid | 1.07 | 0.45 | down |  |
|  | L-Pipecolic Acid | 1.18 | 0.43 | down |  |
|  | Methanesulfonic acid | 1.13 | 0.40 | down |  |
|  | Mevalonic acid | 1.18 | 0.34 | down |  |
|  | 4-Oxopentanoic Acid | 1.14 | 0.28 | down |  |
|  | 2-Phenylpropionic Acid | 1.19 | 0.00 | down |  |
|  | 9-Octadecenoic Acid | 1.19 | 15538.19 | up |  |
|  | Hexanoic Acid | 1.19 | 10174.23 | up |  |
|  | Nonanoic Acid | 1.12 | 0.48 | down |  |
| Others | 6-Methylmercaptopurine | 1.15 | 49.29 | up |  |
|  | 5'-Deoxy-5'-(methylthio)adenosine | 1.19 | 14.14 | up |  |
|  | Xanthine | 1.18 | 9.62 | up |  |
|  | Guanosine 5'-monophosphate | 1.18 | 7.18 | up |  |
|  | 2'-Deoxyguanosine | 1.16 | 4.82 | up |  |
|  | Uridine 5'-monophosphate | 1.14 | 3.58 | up |  |
|  | 8-Azaguanine | 1.17 | 3.51 | up |  |
|  | Isoguanine | 1.19 | 3.45 | up |  |
|  | Thymine | 1.17 | 3.28 | up |  |
|  | Lumazine | 1.18 | 3.07 | up |  |
|  | Guanine | 1.18 | 3.06 | up |  |
|  | 2'-Deoxyadenosine | 1.14 | 2.94 | up |  |
|  | 2'-Deoxycytidine | 1.02 | 2.50 | up |  |
|  | Uridine 5'-diphospho-D-glucose | 1.10 | 2.48 | up |  |
|  | 2'-Deoxyadenosine-5'-monophosphate | 1.08 | 0.47 | down |  |
|  | Succinyladenosine | 1.18 | 0.47 | down |  |
|  | Cyclic 3',5'-Adenylic acid | 1.17 | 0.38 | down |  |
|  | Adenosine 5'-monophosphate | 1.18 | 0.30 | down |  |
|  | Isopentenyladenine-7-N-glucoside | 1.13 | 0.24 | down |  |
|  | Inosine 5'-monophosphate | 1.19 | 0.21 | down |  |
|  | 2'-Deoxyinosine-5'-monophosphate | 1.15 | 0.13 | down |  |
|  | Cytarabine | 1.19 | 0.09 | down |  |
|  | Inosine | 1.18 | 0.06 | down |  |
|  | Cytidine | 1.19 | 0.05 | down |  |
|  | 2-(Dimethylamino)guanosine | 1.18 | 0.04 | down |  |
|  | Adenosine | 1.19 | 0.02 | down |  |
|  | N6-methyladenosine | 1.19 | 0.02 | down |  |
|  | Guanosine | 1.19 | 0.01 | down |  |
|  | Uridine | 1.06 | 0.00 | down |  |
|  | 1-Methyladenosine | 1.19 | 0.00 | down |  |
|  | 2'-O-Methyladenosine | 1.19 | 0.00 | down |  |
|  | 9-(Arabinosyl)hypoxanthine | 1.19 | 0.00 | down |  |
|  | Pentanenitrile, 5-(methylthio)- | 1.08 | 0.50 | down |  |
|  | Benzene, (2-isothiocyanatoethyl)- | 1.14 | 0.40 | down |  |
|  | 1-Butene, 4-isothiocyanato- | 1.12 | 0.27 | down |  |
|  | Berteroin | 1.18 | 0.20 | down |  |
|  | Sulfamide | 1.15 | 0.09 | down |  |
|  | 1-(Phenylthio)isoquinoline | 1.19 | 4384.44 | up |  |
|  | 3-Methyl-pyrrolo(2,3-b)pyrazine | 1.19 | 435.93 | up |  |
|  | 2-n-Propylaziridine | 1.17 | 12.75 | up |  |
|  | 1-(2-furanylmethyl)-1H-Pyrrole | 1.15 | 3.00 | up |  |
|  | 1-Propanone, 1-(2-furanyl)- | 1.09 | 2.44 | up |  |
|  | Furan, 2-pentyl- | 1.11 | 2.19 | up |  |
|  | 2-Thiophenecarboxylic acid, 2-methoxyethyl ester | 1.06 | 0.46 | down |  |
|  | Pyrazine, trimethyl- | 1.11 | 0.29 | down |  |
|  | 4-Acetylaminobiphenyl | 1.19 | 1050.33 | up |  |
|  | 2-Phenylpropenal | 1.19 | 615.43 | up |  |
|  | 3,5-Dimethoxy-4-hydroxytoluene | 1.15 | 2.44 | up |  |
|  | 3-Octanol | 1.19 | 609.49 | up |  |
|  | 1-Pentanol | 1.19 | 20.38 | up |  |
|  | Phenylethyl Alcohol | 1.18 | 12.54 | up |  |
|  | 1-Undecanol | 1.18 | 4.36 | up |  |
|  | (S,Z)-2-Methyl-6-(p-tolyl)hept-2-en-1-ol | 1.15 | 2.13 | up |  |
|  | 2-Ethyl-1-hexanol | 1.18 | 0.34 | down |  |
|  | Coumarin | 1.19 | 11152.04 | up |  |
|  | Pinoresinol* | 1.19 | 3.52 | up |  |
|  | Epipinoresinol* | 1.18 | 3.41 | up |  |
|  | Syringaresinol | 1.18 | 2.06 | up |  |
|  | Pinoresinol-4-O-glucoside | 1.18 | 0.15 | down |  |
|  | 2,5-bis(1,1-dimethylethyl)-1,4-Benzenediol | 1.19 | 292.80 | up |  |
|  | Oxirane, tetradecyl- | 1.19 | 5.52 | up |  |
|  | Methional | 1.19 | 10092.84 | up |  |
|  | BenzeneacetAldehyde | 1.17 | 9.26 | up |  |
|  | 2,4-dihydroxy-6-methyl-BenzAldehyde | 1.14 | 0.35 | down |  |
|  | 1-(1-cyclohexen-1-yl)-1-Propanone | 1.10 | 2.10 | up |  |
|  | 2-Pentadecanone, 6,10,14-trimethyl- | 1.16 | 2.04 | up |  |
|  | Cyanogen bromide | 1.19 | 1122.96 | up |  |
|  | 1-iodo-Octane | 1.18 | 7.33 | up |  |
|  | Phosphoramidous difluoride | 1.13 | 3.50 | up |  |
|  | Hordenine | 1.17 | 0.50 | down |  |
|  | Acetic anhydride | 1.18 | 64143.84 | up |  |
|  | D-Threose | 1.19 | 25845.19 | up |  |
|  | Sinigrin (2-Propenyl Glucosinolate) | 1.19 | 16652.52 | up |  |
|  | 4'-Methoxyresveratrol | 1.19 | 12150.26 | up |  |
|  | 2-[2-(4-Methoxyphenyl)ethyl]chromone | 1.19 | 7668.48 | up |  |
|  | 7-(Methylsulfinyl)Heptyl Glucosinolate | 1.19 | 7113.52 | up |  |
|  | Rhamnose | 1.19 | 5944.52 | up |  |
|  | D-Galactaric acid* | 1.19 | 2567.96 | up |  |
|  | Xylitol | 1.18 | 7.93 | up |  |
|  | 3'-Hydroxy-4'-O-methylglabridin | 1.16 | 6.01 | up |  |
|  | D-Pantothenic Acid | 1.16 | 5.22 | up |  |
|  | D-Erythrose-4-phosphate | 1.17 | 4.13 | up |  |
|  | D-Pinitol | 1.12 | 3.58 | up |  |
|  | D-Glucose 1,6-bisphosphate | 1.18 | 3.43 | up |  |
|  | D-Galacturonic acid* | 1.15 | 3.21 | up |  |
|  | D-Arabitol* | 1.19 | 3.00 | up |  |
|  | D-Glucosamine 1-phosphate | 1.09 | 2.94 | up |  |
|  | Meso-Erythritol | 1.12 | 2.75 | up |  |
|  | Ribitol* | 1.18 | 2.70 | up |  |
|  | D-Glucoronic acid* | 1.18 | 2.54 | up |  |
|  | 6-Methoxy-2-(2-phenylethyl)chromone | 1.10 | 2.48 | up |  |
|  | Sorbitol-6-phosphate | 1.16 | 2.44 | up |  |
|  | D-Glucose 6-phosphate* | 1.15 | 2.42 | up |  |
|  | Glucose-1-phosphate* | 1.16 | 2.40 | up |  |
|  | D-Fructose 6-Phosphate | 1.12 | 2.23 | up |  |
|  | 2'-Aminoacetophenone | 1.17 | 2.19 | up |  |
|  | Norepinephrine | 1.19 | 2.16 | up |  |
|  | Pyridoxine | 1.18 | 2.12 | up |  |
|  | Nicotinate D-ribonucleoside | 1.07 | 2.08 | up |  |
|  | (E)-Cinnamamide | 1.11 | 0.50 | down |  |
|  | Menatetrenone (Vitamin K2) | 1.13 | 0.49 | down |  |
|  | Sucrose-6-phosphate | 1.08 | 0.49 | down |  |
|  | Galloyl-piceid | 1.14 | 0.47 | down |  |
|  | Resveratrol-4'-O--D-(6-O-galloyl)-glucopyranoside | 1.15 | 0.46 | down |  |
|  | Maltotriose | 1.17 | 0.42 | down |  |
|  | 6-Demethoxycapillarisin | 1.04 | 0.40 | down |  |
|  | 5-hydroxymaltol | 1.18 | 0.38 | down |  |
|  | Pyridoxine-5'-O-glucoside | 1.16 | 0.38 | down |  |
|  | Gluconic acid | 1.19 | 0.38 | down |  |
|  | Sulforaphane (4-methylsulphinylbutyl glucosinolate) | 1.18 | 0.31 | down |  |
|  | D-Maltotetraose | 1.18 | 0.28 | down |  |
|  | Maltitol | 1.14 | 0.27 | down |  |
|  | Epigoitrin | 1.19 | 0.18 | down |  |
|  | Inositol* | 1.18 | 0.18 | down |  |
|  | D-Fructose* | 1.18 | 0.18 | down |  |
|  | D-Glucose* | 1.17 | 0.17 | down |  |
|  | D-Galactose* | 1.18 | 0.16 | down |  |
|  | 3-Phenylpropyl Glucosinolate | 1.18 | 0.15 | down |  |
|  | 3-Hydroxy-5-(methylthio)pentyl Glucosinolate | 1.19 | 0.15 | down |  |
|  | D-Melezitose | 1.19 | 0.14 | down |  |
|  | D-Panose* | 1.18 | 0.13 | down |  |
|  | Raffinose* | 1.18 | 0.11 | down |  |
|  | Glucoraphanin | 1.18 | 0.10 | down |  |
|  | 3-Methylsulfinylpropyl glucosinolate | 1.19 | 0.10 | down |  |
|  | 5-Methylthiopentyl glucosinolate (Glucoberteroin) | 1.19 | 0.09 | down |  |
|  | 4-Methylthiobutyl glucosinolate (Glucoerucin) | 1.19 | 0.09 | down |  |
|  | D-Mannose* | 1.18 | 0.09 | down |  |
|  | 8-Methylthiooctyl glucosinolate | 1.18 | 0.08 | down |  |
|  | 2-Hydroxy-2-methylpropylglucosinolate | 1.19 | 0.08 | down |  |
|  | 2(R)-Hydroxy-3-butenyl glucosinolate | 1.19 | 0.08 | down |  |
|  | 1-Methylpropyl glucosinolate | 1.19 | 0.07 | down |  |
|  | 2-Hydroxy-4-Pentenylglucosinolate | 1.19 | 0.05 | down |  |
|  | D-Maltose* | 1.18 | 0.05 | down |  |
|  | Galactinol | 1.16 | 0.05 | down |  |
|  | Stachyose | 1.19 | 0.05 | down |  |
|  | Manninotriose | 1.18 | 0.04 | down |  |
|  | Isomaltulose* | 1.16 | 0.04 | down |  |
|  | D-Trehalose* | 1.18 | 0.04 | down |  |
|  | D-Sucrose* | 1.18 | 0.03 | down |  |
|  | 4-Hydroxyindol-3-ylmethyl glucosinolate | 1.19 | 0.00 | down |  |
|  | 1,6-anhydro--D-glucose | 1.19 | 0.00 | down |  |
|  | 5-Hydroxymethylfurfural | 1.19 | 0.00 | down |  |
|  | Nystose | 1.19 | 0.00 | down |  |
|  | Piperidine | 1.19 | 538692.59 | up |  |
|  | 9-Hydroxysophoramine | 1.19 | 314170.37 | up |  |
|  | Tryptamine | 1.19 | 17747.78 | up |  |
|  | N,N'-Diferuloylputrescine | 1.19 | 11823.93 | up |  |
|  | Methoxyindoleacetic acid | 1.19 | 10409.11 | up |  |
|  | N-Acetylputrescine | 1.19 | 6468.07 | up |  |
|  | N-Benzylmethylene isomethylamine | 1.15 | 50.92 | up |  |
|  | 6-Deoxyfagomine | 1.16 | 50.22 | up |  |
|  | N-(2-Hydroxy-4-methoxyphenyl)acetamide | 1.16 | 40.22 | up |  |
|  | N-benzylformamide | 1.18 | 27.20 | up |  |
|  | 4-Hydroxy-5-(2-oxo-1-pyrrolidinyl)benzoic acid | 1.19 | 22.83 | up |  |
|  | Phenylethanolamine | 1.18 | 17.03 | up |  |
|  | Acetryptine | 1.19 | 12.50 | up |  |
|  | 3-amino-2-naphthoic acid | 1.17 | 9.93 | up |  |
|  | 3-Indoleacrylic acid | 1.17 | 9.44 | up |  |
|  | 1-Methoxy-indole-3-acetamide | 1.17 | 8.90 | up |  |
|  | 4-Coumaroylcholine | 1.19 | 2.69 | up |  |
|  | 1,4-Dihydro-1-Methyl-4-oxo-3-pyridinecarboxamide | 1.18 | 2.63 | up |  |
|  | 5-Hydroxyindole-3-acetic acid | 1.02 | 2.44 | up |  |
|  | Feruloylcholine | 1.16 | 2.36 | up |  |
|  | Phenethylamine | 1.16 | 2.07 | up |  |
|  | Indole-3-cyano-2-O-glucoside | 1.18 | 0.36 | down |  |
|  | Aurantiamide | 1.18 | 0.28 | down |  |
|  | Aurantiamide acetate | 1.17 | 0.20 | down |  |
|  | 10-Formyltetrahydrofolic Acid | 1.19 | 0.12 | down |  |
|  | Caffeoylcholine-4-O-glucoside | 1.19 | 0.10 | down |  |
|  | Feruloylcholine glucoside | 1.19 | 0.09 | down |  |
|  | Indole-3-cyano-6-O-glucoside | 1.19 | 0.07 | down |  |

* denote substances with isomers that are difficult to distinguish by mass spectrometry

Table S4 Differential metabolites of T1 vs T2 in S2

| **Class** | | **Compounds** | | **VIP** | **Fold_Change** | | **Type** | |
| --- | --- | --- | --- | --- | --- | --- | --- | --- |
| Amino acids and their derivatives | | L-Glycyl-L-phenylalanine | 1.21 | | 198588.89 | up |  |  |
|  |  | Hexanoyl-L-glycine | 1.20 | | 76226.30 | up |  |  |
|  |  | L-Phenylalanyl-L-phenylalanine | 1.20 | | 4812.37 | up |  |  |
|  |  | Tryptophan glutamic acid | 1.21 | | 4005.22 | up |  |  |
|  |  | L-Histidine | 1.19 | | 41.45 | up |  |  |
|  |  | L-Lysine-Butanoic Acid | 1.18 | | 30.71 | up |  |  |
|  |  | L-Lysine | 1.15 | | 26.14 | up |  |  |
|  |  | L-Glutamine | 1.15 | | 22.65 | up |  |  |
|  |  | N-Glycyl-L-leucine* | 1.15 | | 21.45 | up |  |  |
|  |  | L-Ornithine | 1.18 | | 21.38 | up |  |  |
|  |  | L-Tyramine | 1.20 | | 21.29 | up |  |  |
|  |  | L-Phenylalanine | 1.20 | | 21.27 | up |  |  |
|  |  | L-Prolyl-L-Phenylalanine | 1.19 | | 19.59 | up |  |  |
|  |  | L-Glycyl-L-isoleucine* | 1.14 | | 19.46 | up |  |  |
|  |  | N6-Acetyl-L-lysine | 1.20 | | 16.56 | up |  |  |
|  |  | L-Valyl-L-Leucine | 1.08 | | 15.45 | up |  |  |
|  |  | L--Glutamyl-L-leucine | 1.18 | | 13.20 | up |  |  |
|  |  | L-Alanyl-L-Phenylalanine | 1.17 | | 10.92 | up |  |  |
|  |  | N-Acetyl-L-tyrosine | 1.19 | | 9.64 | up |  |  |
|  |  | N-Acetyl-L-glycine | 1.20 | | 9.49 | up |  |  |
|  |  | L-Prolyl-L-Leucine | 1.13 | | 9.47 | up |  |  |
|  |  | Glycyl-tryptophan | 1.18 | | 8.90 | up |  |  |
|  |  | L-Tryptophan | 1.20 | | 8.61 | up |  |  |
|  |  | L-Aspartyl-L-Phenylalanine | 1.10 | | 8.57 | up |  |  |
|  |  | L-Valine | 1.19 | | 8.52 | up |  |  |
|  |  | L-Isoleucine* | 1.20 | | 7.81 | up |  |  |
|  |  | L-Norleucine | 1.20 | | 7.71 | up |  |  |
|  |  | L-Leucine* | 1.20 | | 7.42 | up |  |  |
|  |  | L-Alanyl-L-leucine | 1.20 | | 7.31 | up |  |  |
|  |  | cis-4-Hydroxy-D-proline | 1.18 | | 6.98 | up |  |  |
|  |  | L-Leucyl-L-phenylalanine | 1.19 | | 6.24 | up |  |  |
|  |  | -glutamylmethionine | 1.18 | | 6.10 | up |  |  |
|  |  | L-Methionine | 1.18 | | 5.70 | up |  |  |
|  |  | N-Acetyl-L-phenylalanine | 1.20 | | 5.33 | up |  |  |
|  |  | N-Alpha-Acetyl-L-Asparagine | 1.20 | | 4.94 | up |  |  |
|  |  | L-threo-3-Methylaspartate | 1.18 | | 4.86 | up |  |  |
|  |  | N--Acetyl-L-ornithine | 1.18 | | 4.73 | up |  |  |
|  |  | S-(Methyl)glutathione | 1.18 | | 4.70 | up |  |  |
|  |  | N-Acetyl-L-leucine | 1.19 | | 4.38 | up |  |  |
|  |  | L-Tyrosine | 1.02 | | 4.03 | up |  |  |
|  |  | N,N-Dimethylglycine | 1.20 | | 3.69 | up |  |  |
|  |  | S-Methyl-L-cysteine | 1.04 | | 3.61 | up |  |  |
|  |  | N-Acetyl-L-Methionine | 1.20 | | 3.16 | up |  |  |
|  |  | Trimethyllysine | 1.15 | | 3.10 | up |  |  |
|  |  | 4-Hydroxy-L-glutamic acid | 1.15 | | 3.09 | up |  |  |
|  |  | NG,NG-Dimethyl-L-arginine | 1.12 | | 3.08 | up |  |  |
|  |  | N-Acetyl-L-Aspartic Acid | 1.19 | | 2.96 | up |  |  |
|  |  | 3-Hydroxy-3-methylpentane-1,5-dioic acid | 1.16 | | 2.57 | up |  |  |
|  |  | 5-Oxoproline | 1.19 | | 2.54 | up |  |  |
|  |  | 3,4-Dihydroxy-L-phenylalanine (L-Dopa) | 1.07 | | 2.42 | up |  |  |
|  |  | 5-Oxo-L-Proline | 1.13 | | 2.24 | up |  |  |
|  |  | L-Methionine Sulfoximine | 1.17 | | 0.49 | down |  |  |
|  |  | L-Proline | 1.19 | | 0.35 | down |  |  |
|  |  | N-Ethylmaleimide (NEM) | 1.18 | | 0.29 | down |  |  |
|  |  | N-Acetyl-L-Tryptophan | 1.17 | | 0.19 | down |  |  |
|  |  | N-Methyl-Trans-4-Hydroxy-L-Proline | 1.20 | | 0.07 | down |  |  |
|  |  | N-Acetyl-L-Glutamine | 1.21 | | 0.00 | down |  |  |
| Lipids | | LysoPE 18:3 | 1.20 | | 238362.96 | up |  |  |
|  |  | LysoPE 14:0 | 1.12 | | 24.77 | up |  |  |
|  |  | LysoPE 16:1 | 1.09 | | 19.79 | up |  |  |
|  |  | LysoPE 14:0(2n isomer) | 1.09 | | 17.86 | up |  |  |
|  |  | LysoPE 16:0 | 1.18 | | 15.66 | up |  |  |
|  |  | LysoPE 18:3(2n isomer) | 1.14 | | 13.45 | up |  |  |
|  |  | LysoPE 18:0 | 1.20 | | 11.38 | up |  |  |
|  |  | LysoPE 16:1(2n isomer) | 1.05 | | 10.72 | up |  |  |
|  |  | Tetracosanoic Acid (Lignoceric acid) | 1.20 | | 10.12 | up |  |  |
|  |  | 2--Linolenoyl-glycerol-1,3-di-O-glucoside | 1.18 | | 10.00 | up |  |  |
|  |  | LysoPI 16:0 | 1.21 | | 7.95 | up |  |  |
|  |  | Methyl linolenate | 1.19 | | 7.84 | up |  |  |
|  |  | 12-Hydroxyoctadecanoic acid | 1.14 | | 7.67 | up |  |  |
|  |  | 2-Linoleoylglycerol-1,3-di-O-glucoside | 1.18 | | 7.49 | up |  |  |
|  |  | LysoPE 16:0(2n isomer) | 1.08 | | 7.17 | up |  |  |
|  |  | LysoPE 18:1 | 1.20 | | 6.99 | up |  |  |
|  |  | LysoPE 18:0(2n isomer) | 1.20 | | 6.33 | up |  |  |
|  |  | 1-Oleoyl-Sn-Glycerol | 1.14 | | 6.11 | up |  |  |
|  |  | 2-Linoleoylglycerol | 1.19 | | 5.66 | up |  |  |
|  |  | 9-Oxo-12Z-Octadecenoic acid* | 1.20 | | 5.60 | up |  |  |
|  |  | LysoPE 18:1(2n isomer) | 1.16 | | 5.58 | up |  |  |
|  |  | LysoPC 17:2 | 1.06 | | 5.42 | up |  |  |
|  |  | LysoPE 16:3 | 1.06 | | 4.54 | up |  |  |
|  |  | LysoPC 14:0 | 1.18 | | 4.51 | up |  |  |
|  |  | LysoPE 18:2(2n isomer) | 1.10 | | 4.30 | up |  |  |
|  |  | 1--Linolenoyl-glycerol | 1.15 | | 4.13 | up |  |  |
|  |  | 2--Linolenoyl-glycerol | 1.14 | | 4.00 | up |  |  |
|  |  | Oleamide (9-Octadecenamide) | 1.15 | | 3.85 | up |  |  |
|  |  | 1-Eicosanol | 1.20 | | 3.81 | up |  |  |
|  |  | Ricinoleic acid | 1.20 | | 3.79 | up |  |  |
|  |  | 7S,8S-DiHODE; (9Z,12Z)-(7S,8S)  -Dihydroxyoctadeca-  9,12-dienoic acid | 1.20 | | 3.78 | up |  |  |
|  |  | 9,10-DHOME; (12Z)-9,10-Dihydroxyoctadec-12-enoic acid | 1.20 | | 3.58 | up |  |  |
|  |  | Arachidic acid | 1.20 | | 3.49 | up |  |  |
|  |  | LysoPC 18:0 | 1.20 | | 3.47 | up |  |  |
|  |  | LysoPC 19:1 | 1.10 | | 3.43 | up |  |  |
|  |  | LysoPC 16:0(2n isomer) | 1.07 | | 3.41 | up |  |  |
|  |  | LysoPC 17:0(2n isomer) | 1.13 | | 3.32 | up |  |  |
|  |  | 9-Hydroxy-10,12,15-octadecatrienoic acid* | 1.18 | | 2.94 | up |  |  |
|  |  | 3-Hydroxy-palmitic acid methyl ester | 1.17 | | 2.89 | up |  |  |
|  |  | 13-KODE;  (9Z,11E)-13-Oxooctadeca-9,11-dienoic acid* | 1.16 | | 2.86 | up |  |  |
|  |  | 12-Oxo-phytodienoic acid | 1.19 | | 2.81 | up |  |  |
|  |  | 9-Oxo-10E,12Z-octadecadienoic acid | 1.20 | | 2.80 | up |  |  |
|  |  | 9-Hydroperoxy-9Z,11E-Octadecadienoic Acid | 1.20 | | 2.72 | up |  |  |
|  |  | LysoPC 18:0(2n isomer) | 1.18 | | 2.67 | up |  |  |
|  |  | 12,13-DHOME; (9Z)-12,13-Dihydroxyoctadec-9-enoic acid | 1.20 | | 2.63 | up |  |  |
|  |  | LysoPG 16:0 | 1.08 | | 2.54 | up |  |  |
|  |  | LysoPC 17:0 | 1.17 | | 2.51 | up |  |  |
|  |  | 9,10-Epoxyoctadecanoic Acid | 1.20 | | 2.50 | up |  |  |
|  |  | Crepenynic acid | 1.18 | | 2.47 | up |  |  |
|  |  | LysoPC 16:0 | 1.19 | | 2.34 | up |  |  |
|  |  | 13(S)-HODE;13(S)-  Hydroxyoctadeca-9Z,11E-dienoic acid* | 1.16 | | 2.27 | up |  |  |
|  |  | LysoPC 19:2(2n isomer) | 1.01 | | 2.20 | up |  |  |
|  |  | Hexadecanedioic acid | 1.20 | | 2.15 | up |  |  |
|  |  | 9S-Hydroxy-10E,12Z-octadecadienoic acid* | 1.15 | | 2.14 | up |  |  |
|  |  | 4-Hydroxysphinganine | 1.18 | | 2.04 | up |  |  |
|  |  | 1,14-Tetradecanedioic Acid | 1.09 | | 2.03 | up |  |  |
|  |  | Heptadecanoic acid | 1.13 | | 0.49 | down |  |  |
|  |  | Methyl palmitate | 1.16 | | 0.47 | down |  |  |
|  |  | Pentadecanoic Acid | 1.18 | | 0.47 | down |  |  |
|  |  | Tridecanedioic acid | 1.20 | | 0.43 | down |  |  |
|  |  | 5,8,11,14-Pentadecanoamide | 1.17 | | 0.39 | down |  |  |
|  |  | LysoPC 20:2 | 1.17 | | 0.37 | down |  |  |
|  |  | 9,10,11-Trihydroxy-12-octadecenoic acid | 1.19 | | 0.33 | down |  |  |
|  |  | 1-Monomyristin | 1.19 | | 0.30 | down |  |  |
|  |  | Arachidonic Acid | 1.20 | | 0.00 | down |  |  |
|  |  | 1-Linoleoylglycerol | 1.21 | | 0.00 | down |  |  |
|  |  | 17-Hydroxylinolenic acid | 1.20 | | 0.00 | down |  |  |
| Terpenoids | | Maslinic acid* | 1.21 | | 14141.11 | up |  |  |
|  |  | 2,3-Dihydroxy-12-ursen-28-oic acid | 1.20 | | 6413.04 | up |  |  |
|  |  | Corosolic acid* | 1.20 | | 5891.30 | up |  |  |
|  |  | 2,3,23-trihydroxyolean-12-en-28-oic acid | 1.21 | | 5436.30 | up |  |  |
|  |  | 11-Keto-ursolic acid | 1.20 | | 3073.22 | up |  |  |
|  |  | Soyasapogenol B-3-O-glucuronide | 1.20 | | 1781.70 | up |  |  |
|  |  | Soyasaponin IV | 1.20 | | 1765.78 | up |  |  |
|  |  | Soyasapogenol E-3-O-rhamnosyl(1,2)glucosyl(1,2)glucuronide | 1.20 | | 1146.85 | up |  |  |
|  |  | Soyasaponin a (Soyasaponin V) | 1.20 | | 548.87 | up |  |  |
|  |  | Soyasaponin e | 1.00 | | 18.69 | up |  |  |
|  |  | Oleanolic acid | 1.19 | | 10.77 | up |  |  |
|  |  | 30-Norhederagenin | 1.20 | | 9.56 | up |  |  |
|  |  | trans-.beta.-Ionone | 1.19 | | 8.73 | up |  |  |
|  |  | 24,30-Dihydroxy-12(13)-enolupinol | 1.18 | | 7.61 | up |  |  |
|  |  | Betulinic acid | 1.20 | | 6.32 | up |  |  |
|  |  | 12-Hydroxyabietic Acid | 1.17 | | 5.21 | up |  |  |
|  |  | Soyasaponin III | 1.08 | | 4.79 | up |  |  |
|  |  | Soyasaponin b' | 1.04 | | 4.06 | up |  |  |
|  |  | Soyasaponin b (Soyasaponin I) | 1.06 | | 3.89 | up |  |  |
|  |  | Betulonic acid | 1.17 | | 3.81 | up |  |  |
|  |  | Cycloartenol | 1.19 | | 2.32 | up |  |  |
|  |  | Cryptomeridiol | 1.17 | | 0.47 | down |  |  |
| Flavonoids | | Equol; 7,4'-Homoisoflavane | 1.21 | | 16341.48 | up |  |  |
|  |  | Luteolin (5,7,3',4'-Tetrahydroxyflavone) | 1.20 | | 8974.59 | up |  |  |
|  |  | 2'-Hydroxygenistein | 1.20 | | 8799.19 | up |  |  |
|  |  | Calycosin | 1.20 | | 5457.59 | up |  |  |
|  |  | Glycitein | 1.21 | | 4371.96 | up |  |  |
|  |  | Apigenin-8-C-Glucoside (Vitexin) | 1.20 | | 3893.63 | up |  |  |
|  |  | Isoluteolin (Orobol)(5,7,3',4'-tetrahydroxyisoflavone) | 1.20 | | 3815.11 | up |  |  |
|  |  | Luteolin-8-C-glucoside (Orientin) | 1.21 | | 2204.41 | up |  |  |
|  |  | Aromadendrin (Dihydrokaempferol) | 1.17 | | 13.69 | up |  |  |
|  |  | Daidzein | 1.10 | | 10.30 | up |  |  |
|  |  | Apigenin | 1.17 | | 5.10 | up |  |  |
|  |  | Dihydroquercetin(Taxifolin) | 1.09 | | 3.95 | up |  |  |
|  |  | Diosmetin (5,7,3'-Trihydroxy-4'-methoxyflavone) | 1.20 | | 0.45 | down |  |  |
|  |  | Kaempferol-3-O-(2''-acetyl)glucoside | 1.13 | | 0.45 | down |  |  |
|  |  | Luteolin-7-O-(6''-sinapoyl)glucoside | 1.04 | | 0.40 | down |  |  |
|  |  | Kaempferol-3-O-(6''-Sinapyl)glucosyl-(12)-Galactoside | 1.01 | | 0.31 | down |  |  |
|  |  | Catechin | 1.20 | | 0.28 | down |  |  |
|  |  | Kaempferol-3-O-glucoside (Astragalin) | 1.10 | | 0.27 | down |  |  |
|  |  | Diosmetin-7-O-rutinoside (Diosmin) | 1.17 | | 0.26 | down |  |  |
|  |  | Luteolin-7-O-glucoside (Cynaroside) | 1.20 | | 0.25 | down |  |  |
|  |  | Quercetin-3-O-(6''-sinapoyl)glucoside-7-O-rutinoside | 1.18 | | 0.24 | down |  |  |
|  |  | Hispidulin-7-O-(6''-O-p-Coumaroyl)Glucoside | 1.18 | | 0.24 | down |  |  |
|  |  | Kaempferol-4'-O-glucoside | 1.18 | | 0.24 | down |  |  |
|  |  | Tricin-4'-O-glucoside | 1.14 | | 0.23 | down |  |  |
|  |  | Kaempferol-3-O-(2''-sinapoyl)glucosyl-(12)-(6''-malonyl)glucoside | 1.20 | | 0.21 | down |  |  |
|  |  | Epicatechin | 1.16 | | 0.15 | down |  |  |
|  |  | Luteolin-7-O-gentiobioside | 1.17 | | 0.10 | down |  |  |
|  |  | Kaempferol-3-O-sophorotrioside | 1.17 | | 0.03 | down |  |  |
|  |  | Luteolin-7-O-(2'''-O-rhamnosyl)sophoroside-5-O-glucoside | 1.20 | | 0.00 | down |  |  |
|  |  | Hispidulin-7-O-Glucoside | 1.21 | | 0.00 | down |  |  |
|  |  | Quercetin-3-O-sophoroside (Baimaside) | 1.20 | | 0.00 | down |  |  |
|  |  | Tricin-7-O-saccharic acid | 1.20 | | 0.00 | down |  |  |
|  |  | Kaempferol-3-O-galactoside (Trifolin) | 1.20 | | 0.00 | down |  |  |
| Ester | | 2(3H)-Furanone, dihydro-5-(2-octenyl)-, (Z)- | 1.20 | | 246985.17 | up |  |  |
|  |  | Ethyl 9-hexadecenoate | 1.21 | | 9054.02 | up |  |  |
|  |  | Pentanoic acid, 4-methyl-, ethyl ester | 1.20 | | 8290.04 | up |  |  |
|  |  | Benzoic acid, ethyl ester | 1.20 | | 7244.75 | up |  |  |
|  |  | 3-Methylbut-2-enoic acid, 2-methylpentyl ester | 1.21 | | 5547.47 | up |  |  |
|  |  | Butanoic acid, propyl ester | 1.21 | | 5120.55 | up |  |  |
|  |  | (Z)-Ethyl heptadec-9-enoate | 1.21 | | 685.59 | up |  |  |
|  |  | Hexadecanoic acid, propyl ester | 1.17 | | 20.66 | up |  |  |
|  |  | Ethyl Oleate | 1.19 | | 12.22 | up |  |  |
|  |  | trans,trans-9,12-Octadecadienoic acid, propyl ester | 1.18 | | 12.04 | up |  |  |
|  |  | Tetradecanoic acid, ethyl ester | 1.19 | | 8.59 | up |  |  |
|  |  | Octadecanoic acid, ethyl ester | 1.19 | | 7.12 | up |  |  |
|  |  | Pentadecanoic acid, ethyl ester | 1.19 | | 6.61 | up |  |  |
|  |  | Hexanoic acid, propyl ester | 1.15 | | 6.60 | up |  |  |
|  |  | Hexadecanoic acid, ethyl ester | 1.19 | | 5.87 | up |  |  |
|  |  | 9,12-Octadecadienoic acid (Z,Z)-, methyl ester | 1.18 | | 4.80 | up |  |  |
|  |  | 3-Oxobutan-2-yl 2-methylbutanoate | 1.08 | | 4.55 | up |  |  |
|  |  | Benzenepropanoic acid, ethyl ester | 1.05 | | 4.09 | up |  |  |
|  |  | Hexadecanoic acid, butyl ester | 1.20 | | 3.84 | up |  |  |
|  |  | 9-Octadecenoic acid (Z)-, methyl ester | 1.19 | | 3.81 | up |  |  |
|  |  | Hexanoic acid, butyl ester | 1.12 | | 3.30 | up |  |  |
|  |  | Methyl tetradecanoate | 1.05 | | 2.53 | up |  |  |
|  |  | Undecanoic acid, ethyl ester | 1.18 | | 2.35 | up |  |  |
|  |  | 2-Butenoic acid, 3-hexenyl ester, (E,Z)- | 1.18 | | 0.43 | down |  |  |
|  |  | Phenethyl isocyanate | 1.16 | | 0.28 | down |  |  |
|  |  | 2-(2-butoxyethoxy)-Ethanol,acetate | 1.18 | | 0.14 | down |  |  |
|  |  | Butyl benzoate | 1.21 | | 0.00 | down |  |  |
| Organic acids | | Trigallic acid | 1.21 | | 7519.30 | up |  |  |
|  |  | Ethyl caffeate | 1.21 | | 4061.59 | up |  |  |
|  |  | Rosmarinic acid | 1.19 | | 23.67 | up |  |  |
|  |  | Benzamide | 1.20 | | 19.56 | up |  |  |
|  |  | 3-(3-Hydroxyphenyl)-propionic acid | 1.18 | | 11.44 | up |  |  |
|  |  | Hydrocinnamic acid | 1.16 | | 9.17 | up |  |  |
|  |  | Methyl sinapate | 1.20 | | 8.48 | up |  |  |
|  |  | 3-hydroxyphenylacetic acid | 1.16 | | 7.82 | up |  |  |
|  |  | Mucic acid Dimethyl Ester | 1.19 | | 4.99 | up |  |  |
|  |  | Methyl 2,4-dihydroxyphenylacetate | 1.20 | | 4.54 | up |  |  |
|  |  | 2-Hydroxy-3-phenylpropanoic acid | 1.20 | | 3.75 | up |  |  |
|  |  | Ethyl ferulate | 1.19 | | 3.74 | up |  |  |
|  |  | 3-(4-Hydroxyphenyl)-propionic acid* | 1.20 | | 3.56 | up |  |  |
|  |  | 2,6-Dimethoxybenzaldehyde* | 1.20 | | 3.55 | up |  |  |
|  |  | Anthranilate-1-O-Sophoroside | 1.02 | | 3.42 | up |  |  |
|  |  | 3-[(1-Carboxyvinyl)oxy]benzoic acid | 1.19 | | 3.01 | up |  |  |
|  |  | Ferulic acid methyl ester | 1.19 | | 3.01 | up |  |  |
|  |  | Phenethyl caffeate | 1.19 | | 2.96 | up |  |  |
|  |  | 3-Methoxybenzoic acid | 1.18 | | 2.91 | up |  |  |
|  |  | Tyrosol | 1.08 | | 2.33 | up |  |  |
|  |  | 6-O-Caffeoylarbutin | 1.19 | | 2.29 | up |  |  |
|  |  | Benzoic acid | 1.18 | | 2.24 | up |  |  |
|  |  | 4-Aminobenzoic acid | 1.19 | | 2.05 | up |  |  |
|  |  | Sinapic acid | 1.06 | | 2.03 | up |  |  |
|  |  | Vanillin; 4-Hydroxy-3-Methoxybenzaldehyde* | 1.18 | | 0.50 | down |  |  |
|  |  | 4-Methoxyphenylpropionic acid | 1.09 | | 0.47 | down |  |  |
|  |  | Pyrocatechol | 1.20 | | 0.44 | down |  |  |
|  |  | Coniferyl alcohol | 1.17 | | 0.44 | down |  |  |
|  |  | 3-O-Methylgallic acid | 1.18 | | 0.43 | down |  |  |
|  |  | (E)-3-(3,4-dihydroxyphenyl)acrylaldehyde | 1.15 | | 0.39 | down |  |  |
|  |  | Homogentisic acid | 1.19 | | 0.38 | down |  |  |
|  |  | 1-(4-Methoxyphenyl)-1-propanol | 1.18 | | 0.36 | down |  |  |
|  |  | 4-Hydroxybenzoic acid | 1.16 | | 0.35 | down |  |  |
|  |  | 3,4-Dihydroxybenzeneacetic acid | 1.20 | | 0.34 | down |  |  |
|  |  | 4-Nitrophenol | 1.20 | | 0.22 | down |  |  |
|  |  | Salicylic acid | 1.17 | | 0.18 | down |  |  |
|  |  | 2,5-Dihydroxybenzoic acid; Gentisic Acid* | 1.19 | | 0.17 | down |  |  |
|  |  | 6'-O-Sinapoylsucrose | 1.20 | | 0.05 | down |  |  |
|  |  | 6'-O-Feruloyl-D-sucrose | 1.20 | | 0.00 | down |  |  |
|  |  | 1,3-O-Diferuloylglycerol | 1.20 | | 0.00 | down |  |  |
|  |  | Syringaldehyde; 4-Hydroxy-3,5-Dimethoxybenzaldehyde | 1.20 | | 0.00 | down |  |  |
|  |  | 4-O-Glucosyl-sinapate | 1.21 | | 0.00 | down |  |  |
|  |  | Hydroxyphenyllactic acid | 1.21 | | 0.00 | down |  |  |
|  |  | 4-Methylpentanoic acid | 1.20 | | 9608.26 | up |  |  |
|  |  | Valeric acid | 1.20 | | 1238.33 | up |  |  |
|  |  | 5-Aminovaleric acid | 1.16 | | 23.67 | up |  |  |
|  |  | 6-Aminocaproic acid | 1.19 | | 16.48 | up |  |  |
|  |  | 4-Acetamidobutyric acid | 1.20 | | 9.50 | up |  |  |
|  |  | 2-Phenylpropionic Acid | 1.16 | | 7.97 | up |  |  |
|  |  | L-Citramalic acid | 1.20 | | 6.75 | up |  |  |
|  |  | 2-Picolinic acid | 1.20 | | 4.96 | up |  |  |
|  |  | -Hydroxyisovaleric acid | 1.12 | | 4.44 | up |  |  |
|  |  | 5-Acetamidopentanoic Acid | 1.20 | | 4.43 | up |  |  |
|  |  | Argininosuccinic acid | 1.18 | | 4.09 | up |  |  |
|  |  | 2-Hydroxyisocaproic acid | 1.20 | | 4.01 | up |  |  |
|  |  | 3-Hydroxybutyric acid | 1.17 | | 3.89 | up |  |  |
|  |  | -Aminobutyric acid | 1.20 | | 3.85 | up |  |  |
|  |  | 2-Methylsuccinic acid* | 1.20 | | 3.84 | up |  |  |
|  |  | Dimethylmalonic acid* | 1.19 | | 3.72 | up |  |  |
|  |  | 3-Methylmalic acid* | 1.19 | | 3.46 | up |  |  |
|  |  | 3-Methyl-2-Oxobutanoic acid | 1.17 | | 3.18 | up |  |  |
|  |  | 2-Hydroxyglutaric Acid* | 1.18 | | 3.12 | up |  |  |
|  |  | 3-Hydroxypropanoic acid | 1.15 | | 3.10 | up |  |  |
|  |  | Sebacate | 1.04 | | 2.51 | up |  |  |
|  |  | 6-Hydroxyhexanoic acid | 1.19 | | 2.46 | up |  |  |
|  |  | Iminodiacetic acid | 1.12 | | 2.42 | up |  |  |
|  |  | 2-Phosphoglycolate | 1.17 | | 2.37 | up |  |  |
|  |  | L-Malic acid* | 1.17 | | 2.32 | up |  |  |
|  |  | 2-Hydroxyhexadecanoic acid | 1.15 | | 2.17 | up |  |  |
|  |  | 2-Isopropylmalic Acid | 1.05 | | 2.11 | up |  |  |
|  |  | DL-Glyceraldehyde-3-phosphate | 1.10 | | 0.50 | down |  |  |
|  |  | 2-Hydroxyethylphosphonic acid | 1.19 | | 0.11 | down |  |  |
|  |  | Mevalonic acid | 1.20 | | 0.08 | down |  |  |
|  |  | L-Lactic Acid | 1.16 | | 0.06 | down |  |  |
|  |  | Oxalic acid | 1.20 | | 0.05 | down |  |  |
|  |  | Citric Acid | 1.20 | | 0.00 | down |  |  |
|  |  | Butanoic Acid, 2-methyl- | 1.20 | | 177238.15 | up |  |  |
|  |  | 9-Octadecenoic Acid | 1.21 | | 49668.26 | up |  |  |
|  |  | Butanoic Acid, 3-methylbutyl ester | 1.19 | | 26.10 | up |  |  |
|  |  | Nonanoic Acid | 1.13 | | 0.20 | down |  |  |
| Others | | Tryptamine | 1.20 | | 127494.44 | up |  |  |
|  |  | 9-Hydroxysophoramine | 1.20 | | 22892.96 | up |  |  |
|  |  | N,N'-Diferuloylputrescine | 1.20 | | 10956.48 | up |  |  |
|  |  | N-Feruloylserotonin | 1.20 | | 3229.89 | up |  |  |
|  |  | Nicotianamine | 1.20 | | 2386.89 | up |  |  |
|  |  | 3-Hydroxyanthranilic acid | 1.17 | | 52.51 | up |  |  |
|  |  | N-Acetylputrescine | 1.19 | | 37.28 | up |  |  |
|  |  | Phenylethanolamine | 1.20 | | 22.97 | up |  |  |
|  |  | Acetryptine | 1.17 | | 21.93 | up |  |  |
|  |  | N-Benzylmethylene isomethylamine | 1.20 | | 20.62 | up |  |  |
|  |  | Phenethylamine | 1.20 | | 19.61 | up |  |  |
|  |  | 5-Aminolevulinic Acid | 1.19 | | 16.22 | up |  |  |
|  |  | Methoxyindoleacetic acid | 1.19 | | 16.15 | up |  |  |
|  |  | Nicotinic Acid Methyl Ester(Methyl Nicotinate) | 1.20 | | 15.38 | up |  |  |
|  |  | 3-Indoleacrylic acid | 1.20 | | 14.52 | up |  |  |
|  |  | 3-amino-2-naphthoic acid | 1.20 | | 14.36 | up |  |  |
|  |  | Guanidinoacetate | 1.20 | | 11.97 | up |  |  |
|  |  | 1-Methoxy-indole-3-acetamide | 1.20 | | 11.02 | up |  |  |
|  |  | 6-Deoxyfagomine | 1.20 | | 9.77 | up |  |  |
|  |  | Piperidine | 1.19 | | 7.87 | up |  |  |
|  |  | 3-Hydroxypyridine | 1.20 | | 7.07 | up |  |  |
|  |  | Betaine | 1.19 | | 6.85 | up |  |  |
|  |  | 2-(Acetylamino)-3-phenyl-2-propenoic acid | 1.20 | | 4.52 | up |  |  |
|  |  | N-(2-Hydroxy-4-methoxyphenyl)acetamide | 1.04 | | 4.29 | up |  |  |
|  |  | 4-Hydroxy-5-(2-oxo-1-pyrrolidinyl)benzoic acid | 1.04 | | 3.80 | up |  |  |
|  |  | N-benzylformamide | 1.05 | | 3.66 | up |  |  |
|  |  | Feruloylcholine | 1.20 | | 3.32 | up |  |  |
|  |  | 4-Coumaroylcholine | 1.20 | | 2.62 | up |  |  |
|  |  | N-Feruloyltyramine | 1.15 | | 2.43 | up |  |  |
|  |  | Aurantiamide | 1.21 | | 0.23 | down |  |  |
|  |  | 10-Formyltetrahydrofolic Acid | 1.20 | | 0.16 | down |  |  |
|  |  | Aurantiamide acetate | 1.20 | | 0.08 | down |  |  |
|  |  | Caffeoylcholine-4-O-glucoside | 1.20 | | 0.00 | down |  |  |
|  |  | Indole-3-cyano-2-O-glucoside | 1.20 | | 0.00 | down |  |  |
|  |  | Indole-3-cyano-6-O-glucoside | 1.20 | | 0.00 | down |  |  |
|  |  | Feruloylcholine glucoside | 1.20 | | 0.00 | down |  |  |
|  |  | 2'-Deoxyadenosine-5'-monophosphate | 1.21 | | 3886.33 | up |  |  |
|  |  | 2'-Deoxycytidine | 1.20 | | 2565.00 | up |  |  |
|  |  | Uridine 5'-diphosphate | 1.20 | | 1056.77 | up |  |  |
|  |  | 6-Methylmercaptopurine | 1.20 | | 21.16 | up |  |  |
|  |  | 5'-Deoxy-5'-(methylthio)adenosine | 1.19 | | 13.89 | up |  |  |
|  |  | Flavin Single Nucleotide(FMN) | 1.13 | | 4.61 | up |  |  |
|  |  | Cytarabine | 1.17 | | 4.51 | up |  |  |
|  |  | Adenosine 5'-monophosphate | 1.09 | | 4.20 | up |  |  |
|  |  | 1-Methylxanthine | 1.06 | | 4.18 | up |  |  |
|  |  | Adenosine | 1.05 | | 4.15 | up |  |  |
|  |  | Inosine 5'-monophosphate | 1.02 | | 3.37 | up |  |  |
|  |  | 2-(Dimethylamino)guanosine | 1.03 | | 2.93 | up |  |  |
|  |  | Guanosine | 1.04 | | 2.93 | up |  |  |
|  |  | Nicotinic acid adenine dinucleotide | 1.19 | | 2.71 | up |  |  |
|  |  | Succinyladenosine | 1.20 | | 2.71 | up |  |  |
|  |  | Thymine | 1.20 | | 2.30 | up |  |  |
|  |  | 8-Azaguanine | 1.16 | | 0.23 | down |  |  |
|  |  | Guanine | 1.09 | | 0.23 | down |  |  |
|  |  | Isoguanine | 1.15 | | 0.22 | down |  |  |
|  |  | Hypoxanthine | 1.19 | | 0.21 | down |  |  |
|  |  | 2'-Deoxyinosine-5'-monophosphate | 1.20 | | 0.13 | down |  |  |
|  |  | Cyclic 3',5'-Adenylic acid | 1.19 | | 0.04 | down |  |  |
|  |  | Guanosine 3',5'-cyclic monophosphate | 1.20 | | 0.01 | down |  |  |
|  |  | Dimethyl triSulfur compounds | 1.17 | | 3.40 | up |  |  |
|  |  | Furan, 2-[(methyldithio)methyl]- | 1.14 | | 2.52 | up |  |  |
|  |  | Pentanenitrile, 5-(methylthio)- | 1.15 | | 2.03 | up |  |  |
|  |  | Berteroin | 1.18 | | 0.29 | down |  |  |
|  |  | Benzene, (2-isothiocyanatoethyl)- | 1.20 | | 0.18 | down |  |  |
|  |  | 1-Butene, 4-isothiocyanato- | 1.20 | | 0.12 | down |  |  |
|  |  | Sulfamide | 1.17 | | 0.07 | down |  |  |
|  |  | 1-(Phenylthio)isoquinoline | 1.21 | | 409.00 | up |  |  |
|  |  | 2,6-pyridinedicarboxamide | 1.14 | | 2.16 | up |  |  |
|  |  | 1-(2-furanylmethyl)-1H-Pyrrole | 1.20 | | 4.80 | up |  |  |
|  |  | isopropenyl-Pyrazine | 1.15 | | 3.61 | up |  |  |
|  |  | Indole | 1.17 | | 41.35 | up |  |  |
|  |  | 1,4-Dihydro-4-oxopyridazine | 1.20 | | 3.47 | up |  |  |
|  |  | 2-n-Propylaziridine | 1.19 | | 46.99 | up |  |  |
|  |  | Pyrazine | 1.13 | | 0.30 | down |  |  |
|  |  | 2-Phenylpropenal | 1.20 | | 3053.76 | up |  |  |
|  |  | 2-methoxy-Phenol | 1.15 | | 7.94 | up |  |  |
|  |  | Phenol, 4-ethyl- | 1.17 | | 3.02 | up |  |  |
|  |  | Phenylethyl Alcohol | 1.19 | | 33.93 | up |  |  |
|  |  | 1-Hexadecanol | 1.15 | | 15.85 | up |  |  |
|  |  | 3-Phenylpropanol | 1.12 | | 13.36 | up |  |  |
|  |  | Coumarin | 1.20 | | 5493.56 | up |  |  |
|  |  | Fraxidin (8-Hydroxy-6,7-dimethoxycoumarin) | 1.19 | | 2.78 | up |  |  |
|  |  | Isofraxidin | 1.20 | | 2.51 | up |  |  |
|  |  | 5,7-Dihydroxy-4-methylcoumarin | 1.19 | | 0.10 | down |  |  |
|  |  | Epipinoresinol* | 1.18 | | 0.10 | down |  |  |
|  |  | Pinoresinol* | 1.18 | | 0.09 | down |  |  |
|  |  | Pinoresinol-4-O-glucoside | 1.20 | | 0.00 | down |  |  |
|  |  | 1-isocyano-3-methyl-Benzene | 1.17 | | 0.42 | down |  |  |
|  |  | 2,5-bis(1,1-dimethylethyl)-1,4-Benzenediol | 1.20 | | 594.55 | up |  |  |
|  |  | 1-Octadecene | 1.20 | | 2406.82 | up |  |  |
|  |  | Oxirane, tetradecyl- | 1.18 | | 4.71 | up |  |  |
|  |  | BenzeneacetAldehyde | 1.16 | | 6.45 | up |  |  |
|  |  | Methional | 1.13 | | 3.72 | up |  |  |
|  |  | 2-Pentadecanone | 1.15 | | 3.26 | up |  |  |
|  |  | Ethanone, 1-(2-hydroxy-5-methylphenyl)- | 1.10 | | 2.06 | up |  |  |
|  |  | 1-iodo-Octane | 1.07 | | 20.14 | up |  |  |
|  |  | Cyanogen bromide | 1.20 | | 0.00 | down |  |  |
|  |  | D-Xylonic acid | 1.20 | | 156059.26 | up |  |  |
|  |  | 1,5-Anhydro-D-glucitol | 1.21 | | 17151.85 | up |  |  |
|  |  | 3-Phenylpropyl Glucosinolate | 1.21 | | 2411.19 | up |  |  |
|  |  | 1-Methylpropyl glucosinolate | 1.21 | | 2275.04 | up |  |  |
|  |  | D-Threose | 1.20 | | 25.08 | up |  |  |
|  |  | D-Saccharic acid* | 1.19 | | 14.34 | up |  |  |
|  |  | D-Pinitol | 1.19 | | 7.50 | up |  |  |
|  |  | 5-Hydroxy-6-methoxy-2-(2-phenylethyl)chromone | 1.18 | | 5.63 | up |  |  |
|  |  | D-Glucosamine 1-phosphate | 1.18 | | 5.47 | up |  |  |
|  |  | 2-[2-(4-Methoxyphenyl)ethyl]chromone | 1.17 | | 3.67 | up |  |  |
|  |  | 6-Methoxy-2-(2-phenylethyl)chromone | 1.20 | | 3.58 | up |  |  |
|  |  | trans-Trimethoxyresveratrol | 1.20 | | 3.36 | up |  |  |
|  |  | 2-Dehydro-3-deoxy-L-arabinonate | 1.18 | | 3.26 | up |  |  |
|  |  | Pyridoxine-5'-O-glucoside | 1.18 | | 3.25 | up |  |  |
|  |  | 3-Dehydro-L-Threonic Acid* | 1.12 | | 3.01 | up |  |  |
|  |  | 2'-Aminoacetophenone | 1.17 | | 2.31 | up |  |  |
|  |  | D-Arabinono-1,4-lactone | 1.08 | | 2.27 | up |  |  |
|  |  | Sinigrin (2-Propenyl Glucosinolate) | 1.18 | | 2.25 | up |  |  |
|  |  | Orotic acid (Vitamin B13) | 1.18 | | 2.20 | up |  |  |
|  |  | 4-Methylsulfonyl-3-butenyl Glucosinolate | 1.03 | | 2.17 | up |  |  |
|  |  | Sorbitol-6-phosphate | 1.18 | | 2.08 | up |  |  |
|  |  | Biotin | 1.18 | | 2.07 | up |  |  |
|  |  | L-Ascorbic acid (Vitamin C) | 1.17 | | 0.48 | down |  |  |
|  |  | Raffinose* | 1.14 | | 0.47 | down |  |  |
|  |  | Resveratrol-4'-O--D-(6-O-galloyl)-glucopyranoside | 1.17 | | 0.41 | down |  |  |
|  |  | Galloyl-piceid | 1.20 | | 0.38 | down |  |  |
|  |  | 5-hydroxymaltol | 1.20 | | 0.33 | down |  |  |
|  |  | D-Maltotetraose | 1.17 | | 0.30 | down |  |  |
|  |  | D-Threitol | 1.19 | | 0.30 | down |  |  |
|  |  | Epigoitrin | 1.20 | | 0.16 | down |  |  |
|  |  | 2,3-Dihydroxypropanal | 1.19 | | 0.14 | down |  |  |
|  |  | Sulforaphane (4-methylsulphinylbutyl glucosinolate) | 1.19 | | 0.06 | down |  |  |
|  |  | 4-Methyl-5-thiazoleethanol | 1.20 | | 0.06 | down |  |  |
|  |  | 8-Methylthiooctyl glucosinolate | 1.20 | | 0.00 | down |  |  |
|  |  | 4-hydroxyphenyl acrylaldehyde | 1.21 | | 0.00 | down |  |  |
|  |  | Maltitol | 1.20 | | 0.00 | down |  |  |
|  |  | 4-Ketopinoresinol | 1.21 | | 0.00 | down |  |  |
|  |  | N-(beta-D-Glucosyl)nicotinate | 1.20 | | 0.00 | down |  |  |
|  |  | Glucoraphanin | 1.21 | | 0.00 | down |  |  |
|  |  | 3-Hydroxy-5-(methylthio)pentyl Glucosinolate | 1.21 | | 0.00 | down |  |  |

* denote substances with isomers that are difficult to distinguish by mass spectrometry

Table S5 Differential metabolites of T1 vs T2 in S3

| **Class** | | **Compounds** | | **VIP** | **Fold_Change Type** | | |
| --- | --- | --- | --- | --- | --- | --- | --- |
| Amino acids and their derivatives | | L-Prolyl-L-Phenylalanine | 1.22 | | 126014.81 | up | |
|  |  | Hexanoyl-L-glycine | 1.22 | | 9130.33 | up | |
|  |  | L-Phenylalanyl-L-phenylalanine | 1.22 | | 2942.56 | up | |
|  |  | L-Alanine | 1.22 | | 2186.41 | up | |
|  |  | L-Valyl-L-Leucine | 1.19 | | 61.05 | up | |
|  |  | L-Phenylalanine | 1.21 | | 21.85 | up | |
|  |  | L-Tyramine | 1.22 | | 19.37 | up | |
|  |  | L-Glycyl-L-isoleucine* | 1.20 | | 15.38 | up | |
|  |  | N-Glycyl-L-leucine* | 1.21 | | 14.15 | up | |
|  |  | L-Valine | 1.22 | | 11.44 | up | |
|  |  | L-Leucine* | 1.21 | | 10.41 | up | |
|  |  | L-Isoleucine* | 1.21 | | 9.94 | up | |
|  |  | L-Norleucine | 1.22 | | 9.76 | up | |
|  |  | L-Lysine-Butanoic Acid | 1.19 | | 8.78 | up | |
|  |  | L-Tryptophan | 1.21 | | 8.45 | up | |
|  |  | N6-Acetyl-L-lysine | 1.21 | | 6.41 | up | |
|  |  | L-Alanyl-L-Phenylalanine | 1.21 | | 5.84 | up | |
|  |  | cis-4-Hydroxy-D-proline | 1.20 | | 5.67 | up | |
|  |  | N-Methylglycine | 1.15 | | 5.18 | up | |
|  |  | N,N-Dimethylglycine | 1.21 | | 4.71 | up | |
|  |  | L-Aspartyl-L-Phenylalanine | 1.21 | | 4.37 | up | |
|  |  | L-Methionine | 1.06 | | 4.37 | up | |
|  |  | N-Alpha-Acetyl-L-Asparagine | 1.18 | | 4.13 | up | |
|  |  | L-Alanyl-L-leucine | 1.21 | | 3.58 | up | |
|  |  | N-Acetyl-L-leucine | 1.18 | | 3.10 | up | |
|  |  | L-Alanyl-L-Alanine | 1.18 | | 3.04 | up | |
|  |  | L-Tyrosine | 1.17 | | 2.92 | up | |
|  |  | S-Methyl-L-cysteine | 1.17 | | 2.88 | up | |
|  |  | N-Acetyl-L-Methionine | 1.12 | | 2.87 | up | |
|  |  | N--Acetyl-L-ornithine | 1.13 | | 2.74 | up | |
|  |  | L-Methionine Sulfoximine | 1.21 | | 2.72 | up | |
|  |  | NG,NG-Dimethyl-L-arginine | 1.17 | | 2.49 | up | |
|  |  | L--Glutamyl-L-leucine | 1.13 | | 2.31 | up | |
|  |  | L-Lysine | 1.10 | | 2.10 | up | |
|  |  | L-Cyclopentylglycine | 1.17 | | 0.50 | down | |
|  |  | N-Acetyl-L-threonine | 1.11 | | 0.49 | down | |
|  |  | O-Acetylserine | 1.21 | | 0.31 | down | |
|  |  | L-Serine | 1.21 | | 0.27 | down | |
|  |  | L-Proline | 1.11 | | 0.19 | down | |
|  |  | N-Acetyl-L-Tryptophan | 1.15 | | 0.18 | down | |
|  |  | L-Glutamic acid | 1.22 | | 0.16 | down | |
|  |  | N-Methyl-Trans-4-Hydroxy-L-Proline | 1.22 | | 0.04 | down | |
|  |  | -Glutamyltyrosine | 1.22 | | 0.00 | down | |
| Lipids | | 9,12,13-Trihydroxy-10,15-octadecadienoic acid | 1.22 | | 6070.04 | up | |
|  |  | 9,10-DHOME; (12Z)-9,10-Dihydroxyoctadec-12-enoic acid | 1.22 | | 32.36 | up | |
|  |  | 12-Hydroxyoctadecanoic acid | 1.17 | | 24.24 | up | |
|  |  | Methyl linolenate | 1.08 | | 7.05 | up | |
|  |  | Tetracosanoic Acid (Lignoceric acid) | 1.19 | | 6.92 | up | |
|  |  | LysoPE 17:1 | 1.07 | | 5.74 | up | |
|  |  | Ricinoleic acid | 1.21 | | 5.51 | up | |
|  |  | 1-Eicosanol | 1.20 | | 5.48 | up | |
|  |  | LysoPE 16:0(2n isomer) | 1.10 | | 4.27 | up | |
|  |  | LysoPE 18:0 | 1.21 | | 4.26 | up | |
|  |  | 9-Hydroxy-10,12,15-octadecatrienoic acid* | 1.16 | | 3.94 | up | |
|  |  | 13-KODE; (9Z,11E)-13-Oxooctadeca-9,11-dienoic acid* | 1.16 | | 3.90 | up | |
|  |  | 13(S)-HODE;13(S)-Hydroxyoctadeca-9Z,11E-dienoic acid* | 1.17 | | 3.69 | up | |
|  |  | 9S-Hydroxy-10E,12Z-octadecadienoic acid* | 1.17 | | 3.54 | up | |
|  |  | 7S,8S-DiHODE; (9Z,12Z)-(7S,8S)-Dihydroxyoctadeca-9,12-dienoic acid | 1.21 | | 3.42 | up | |
|  |  | LysoPI 16:0 | 1.10 | | 3.32 | up | |
|  |  | 9-Hydroperoxy-9Z,11E-Octadecadienoic Acid | 1.21 | | 3.29 | up | |
|  |  | 9,10,13-Trihydroxy-11-Octadecenoic Acid | 1.21 | | 3.01 | up | |
|  |  | LysoPE 18:0(2n isomer) | 1.16 | | 2.86 | up | |
|  |  | LysoPE 18:1 | 1.20 | | 2.77 | up | |
|  |  | LysoPE 16:1 | 1.03 | | 2.68 | up | |
|  |  | 12,13-Epoxy-9-Octadecenoic Acid | 1.12 | | 2.63 | up | |
|  |  | LysoPE 18:2(2n isomer) | 1.01 | | 2.60 | up | |
|  |  | 13-methylmyristic acid | 1.18 | | 2.59 | up | |
|  |  | 13S-Hydroperoxy-9Z,11E-octadecadienoic acid | 1.20 | | 2.58 | up | |
|  |  | LysoPE 16:0 | 1.12 | | 2.57 | up | |
|  |  | LysoPG 16:0 | 1.06 | | 2.54 | up | |
|  |  | 3-Hydroxy-palmitic acid methyl ester | 1.18 | | 2.50 | up | |
|  |  | LysoPC 16:0(2n isomer) | 1.05 | | 2.49 | up | |
|  |  | Arachidic acid | 1.17 | | 2.48 | up | |
|  |  | Hexadecanedioic acid | 1.21 | | 2.31 | up | |
|  |  | 12,13-DHOME; (9Z)-12,13-Dihydroxyoctadec-9-enoic acid | 1.22 | | 2.28 | up | |
|  |  | 9-Oxo-10E,12Z-octadecadienoic acid | 1.17 | | 2.23 | up | |
|  |  | LysoPE 14:0 | 1.00 | | 2.23 | up | |
|  |  | LysoPC 17:2 | 1.02 | | 2.14 | up | |
|  |  | 9,10-Dihydroxy-12,13-epoxyoctadecanoic acid | 1.16 | | 2.10 | up | |
|  |  | Hydroxy ricinoleic acid | 1.21 | | 2.10 | up | |
|  |  | Octanoic acid | 1.16 | | 2.06 | up | |
|  |  | Crepenynic acid | 1.21 | | 2.04 | up | |
|  |  | 5,8,11,14-Pentadecanoamide | 1.14 | | 0.50 | down | |
|  |  | LysoPC 18:3(2n isomer) | 1.03 | | 0.48 | down | |
|  |  | 1-Monomyristin | 1.15 | | 0.40 | down | |
|  |  | Methyl palmitate | 1.16 | | 0.39 | down | |
|  |  | 2R-hydroxy-9Z,12Z,15Z-octadecatrienoic acid | 1.20 | | 0.35 | down | |
|  |  | Choline Alfoscerate | 1.20 | | 0.29 | down | |
|  |  | LysoPC 20:0 | 1.21 | | 0.14 | down | |
|  |  | LysoPC 20:2 | 1.21 | | 0.11 | down | |
|  |  | Arachidonic Acid | 1.22 | | 0.00 | down | |
|  |  | 1-Linoleoylglycerol | 1.22 | | 0.00 | down | |
|  |  | 17-Hydroxylinolenic acid | 1.22 | | 0.00 | down | |
| Terpenoids | | Maslinic acid* | 1.22 | | 14976.67 | up | |
|  |  | 2,3-Dihydroxy-12-ursen-28-oic acid | 1.22 | | 6199.89 | up | |
|  |  | Corosolic acid* | 1.22 | | 6056.67 | up | |
|  |  | Soyasaponin c | 1.22 | | 4460.81 | up | |
|  |  | Soyasaponin e | 1.21 | | 1357.50 | up | |
|  |  | Soyasapogenol B-3-O-glucuronide | 1.22 | | 1256.83 | up | |
|  |  | Soyasaponin IV | 1.22 | | 1035.41 | up | |
|  |  | Soyasaponin III | 1.20 | | 7.92 | up | |
|  |  | Soyasaponin b' | 1.20 | | 6.50 | up | |
|  |  | Soyasaponin b (Soyasaponin I) | 1.16 | | 5.80 | up | |
|  |  | 30-Norhederagenin | 1.21 | | 5.29 | up | |
|  |  | Betulonic acid | 1.16 | | 4.51 | up | |
|  |  | 3-Cyclohexen-1-ol, 4-methyl-1-(1-methylethyl)-, (R)- | 1.19 | | 3.12 | up | |
|  |  | Cycloartenol | 1.20 | | 3.00 | up | |
|  |  | trans-.beta.-Ionone | 1.19 | | 2.62 | up | |
|  |  | Cryptomeridiol | 1.19 | | 0.23 | down | |
| Flavonoids | | Genistein | 1.22 | | 9176.67 | up | |
|  |  | 2'-Hydroxygenistein | 1.22 | | 8504.52 | up | |
|  |  | Luteolin (5,7,3',4'-Tetrahydroxyflavone) | 1.22 | | 8195.30 | up | |
|  |  | 3',4',7-Trihydroxyflavone | 1.22 | | 6254.74 | up | |
|  |  | Isoluteolin (Orobol)(5,7,3',4'-tetrahydroxyisoflavone) | 1.22 | | 3655.93 | up | |
|  |  | Equol; 7,4'-Homoisoflavane | 1.22 | | 3517.52 | up | |
|  |  | Calycosin | 1.22 | | 2976.00 | up | |
|  |  | Luteolin-8-C-glucoside (Orientin) | 1.22 | | 2911.96 | up | |
|  |  | Glycitein | 1.22 | | 2699.30 | up | |
|  |  | Apigenin-7-O-(6''-acetyl)glucoside | 1.22 | | 589.64 | up | |
|  |  | Aromadendrin (Dihydrokaempferol) | 1.18 | | 42.80 | up | |
|  |  | 3',7-dihydroxy-4'-methoxyflavone | 1.19 | | 10.54 | up | |
|  |  | Genistein-8-C-glucoside | 1.14 | | 6.17 | up | |
|  |  | Dihydroquercetin(Taxifolin) | 1.21 | | 5.81 | up | |
|  |  | Daidzein | 1.11 | | 5.23 | up | |
|  |  | Apigenin | 1.19 | | 4.38 | up | |
|  |  | Biochanin A | 1.20 | | 3.37 | up | |
|  |  | Kaempferol-4'-O-glucoside | 1.03 | | 0.49 | down | |
|  |  | Diosmetin (5,7,3'-Trihydroxy-4'-methoxyflavone) | 1.21 | | 0.49 | down | |
|  |  | Kaempferol-3-O-glucoside (Astragalin) | 1.19 | | 0.48 | down | |
|  |  | Quercetin-3-O-rhamnoside(Quercitrin) | 1.11 | | 0.47 | down | |
|  |  | Quercetin-3-O-(6''-sinapoyl)glucoside-7-O-rutinoside | 1.12 | | 0.45 | down | |
|  |  | Luteolin-7-O-glucoside (Cynaroside) | 1.20 | | 0.41 | down | |
|  |  | Kaempferol-3-O-(2''-sinapoyl)glucosyl-(12)-(6''-acetyl)glucoside | 1.14 | | 0.37 | down | |
|  |  | Kaempferol-3-O-(2''-sinapoyl)glucosyl-(12)-(6''-malonyl)glucoside | 1.22 | | 0.35 | down | |
|  |  | Kaempferol-3-O-(2''-acetyl)glucoside | 1.19 | | 0.27 | down | |
|  |  | Diosmetin-7-O-rutinoside (Diosmin) | 1.18 | | 0.24 | down | |
|  |  | Epicatechin | 1.20 | | 0.23 | down | |
|  |  | Kaempferol-3-O-sophorotrioside | 1.13 | | 0.21 | down | |
|  |  | Tricin-4'-O-glucoside | 1.22 | | 0.00 | down | |
|  |  | Tricin-7-O-saccharic acid | 1.22 | | 0.00 | down | |
|  |  | Kaempferol-3-O-galactoside (Trifolin) | 1.22 | | 0.00 | down | |
| Ester | | Ethyl 9-hexadecenoate | 1.22 | | 17678.31 | up | |
|  |  | Butanoic acid, 3-methyl-, 2-methylbutyl ester | 1.21 | | 12022.68 | up | |
|  |  | Pentanoic acid, 4-methyl-, ethyl ester | 1.21 | | 4671.00 | up | |
|  |  | (Z)-Ethyl heptadec-9-enoate | 1.22 | | 2187.04 | up | |
|  |  | n-Capric acid isobutyl ester | 1.22 | | 1154.81 | up | |
|  |  | N-(3-Iodopropyl)carbamic acid, benzyl ester | 1.22 | | 1086.41 | up | |
|  |  | n-Butyl laurate | 1.22 | | 670.04 | up | |
|  |  | 3-Phenylpropionic acid, 3-pentyl ester | 1.21 | | 421.03 | up | |
|  |  | Hexadecanoic acid, propyl ester | 1.22 | | 308.62 | up | |
|  |  | Butanoic acid, propyl ester | 1.20 | | 135.74 | up | |
|  |  | Butanoic acid, butyl ester | 1.21 | | 124.96 | up | |
|  |  | trans,trans-9,12-Octadecadienoic acid, propyl ester | 1.20 | | 90.04 | up | |
|  |  | Octadecanoic acid, ethyl ester | 1.21 | | 87.86 | up | |
|  |  | Ethyl Oleate | 1.21 | | 63.03 | up | |
|  |  | Hexadecanoic acid, ethyl ester | 1.21 | | 59.94 | up | |
|  |  | Tetradecanoic acid, ethyl ester | 1.22 | | 55.05 | up | |
|  |  | Pentadecanoic acid, ethyl ester | 1.21 | | 49.37 | up | |
|  |  | Hexadecanoic acid, butyl ester | 1.21 | | 48.48 | up | |
|  |  | Undecanoic acid, ethyl ester | 1.22 | | 23.62 | up | |
|  |  | Isopropyl palmitate | 1.04 | | 21.21 | up | |
|  |  | Benzenepropanoic acid, ethyl ester | 1.17 | | 19.23 | up | |
|  |  | 9,12-Octadecadienoic acid (Z,Z)-, methyl ester | 1.18 | | 9.57 | up | |
|  |  | Butanoic acid, octyl ester | 1.20 | | 8.38 | up | |
|  |  | Hexanoic acid, propyl ester | 1.16 | | 7.84 | up | |
|  |  | 9-Octadecenoic acid (Z)-, methyl ester | 1.18 | | 6.97 | up | |
|  |  | Hexanoic acid, butyl ester | 1.07 | | 6.14 | up | |
|  |  | Methyl tetradecanoate | 1.21 | | 4.02 | up | |
|  |  | Methyl stearate | 1.15 | | 3.88 | up | |
|  |  | 3-Oxobutan-2-yl 2-methylbutanoate | 1.15 | | 2.74 | up | |
|  |  | Benzoic acid, ethyl ester | 1.07 | | 2.49 | up | |
|  |  | (3S,3aR)-3-Butyl-3a,4,5,6-tetrahydroisobenzofuran-1(3H)-one | 1.16 | | 2.16 | up | |
|  |  | Propanoic acid, 2-methyl-, 3-phenylpropyl ester | 1.08 | | 2.08 | up | |
|  |  | 2-Methoxy-5-methylphenol, acetate | 1.11 | | 0.25 | down | |
|  |  | 2-(2-butoxyethoxy)-Ethanol,acetate | 1.18 | | 0.16 | down | |
| Organic acids | | Hexanoic Acid | 1.22 | | 4133801.39 | up | |
|  |  | Butanoic Acid, 2-methyl- | 1.22 | | 203432.20 | up | |
|  |  | Benzeneacetic acid | 1.22 | | 63969.70 | up | |
|  |  | 9-Octadecenoic Acid | 1.22 | | 7566.22 | up | |
|  |  | Butanoic Acid, 3-methylbutyl ester | 1.21 | | 40.54 | up | |
|  |  | Butanoic Acid, 3-methyl- | 1.17 | | 32.62 | up | |
|  |  | Nonanoic Acid | 1.09 | | 0.38 | down | |
|  |  | 4-Methylpentanoic acid | 1.22 | | 1304.79 | up | |
|  |  | 5-Aminovaleric acid | 1.21 | | 36.20 | up | |
|  |  | 6-Aminocaproic acid | 1.21 | | 23.42 | up | |
|  |  | 2-Phenylpropionic Acid | 1.20 | | 10.18 | up | |
|  |  | Iminodiacetic acid | 1.22 | | 8.87 | up | |
|  |  | Argininosuccinic acid | 1.12 | | 6.91 | up | |
|  |  | -Aminobutyric acid | 1.22 | | 5.01 | up | |
|  |  | 2-Hydroxybutyric Acid* | 1.10 | | 3.46 | up | |
|  |  | Succinic anhydride | 1.14 | | 3.19 | up | |
|  |  | 2-Hydroxyisobutyric acid* | 1.17 | | 3.09 | up | |
|  |  | Decanoic acid | 1.20 | | 3.00 | up | |
|  |  | 2-Phosphoglycolate | 1.17 | | 2.88 | up | |
|  |  | 2-Hydroxyisocaproic acid | 1.20 | | 2.80 | up | |
|  |  | 5-Acetamidopentanoic Acid | 1.18 | | 2.42 | up | |
|  |  | 2-Methylsuccinic acid* | 1.19 | | 2.32 | up | |
|  |  | Succinic acid* | 1.18 | | 2.27 | up | |
|  |  | Aminomalonic acid | 1.17 | | 2.25 | up | |
|  |  | -Hydroxyisovaleric acid | 1.10 | | 2.19 | up | |
|  |  | Dimethylmalonic acid* | 1.20 | | 2.14 | up | |
|  |  | L-Citramalic acid | 1.09 | | 2.10 | up | |
|  |  | 4,8-Dihydroxyquinoline-2-carboxylic acid | 1.21 | | 0.50 | down | |
|  |  | 2-Isopropylmalic Acid | 1.06 | | 0.48 | down | |
|  |  | DL-Glyceraldehyde-3-phosphate | 1.11 | | 0.47 | down | |
|  |  | L-Pipecolic Acid | 1.18 | | 0.46 | down | |
|  |  | Tartronate semialdehyde* | 1.19 | | 0.46 | down | |
|  |  | Hydroxypyruvic acid* | 1.18 | | 0.41 | down | |
|  |  | 3-Methyl-2-Oxobutanoic acid | 1.15 | | 0.37 | down | |
|  |  | Methanesulfonic acid | 1.22 | | 0.22 | down | |
|  |  | Citric Acid | 1.14 | | 0.19 | down | |
|  |  | Ethyl caffeate | 1.22 | | 13204.44 | up | |
|  |  | Trigallic acid | 1.22 | | 6496.33 | up | |
|  |  | Vanillic Acid-4-O-Glucuronide | 1.22 | | 4815.30 | up | |
|  |  | 1-O-Caffeoyl--D-glucose | 1.22 | | 4812.89 | up | |
|  |  | Mucic acid Dimethyl Ester | 1.22 | | 1233.00 | up | |
|  |  | Rosmarinic acid | 1.22 | | 42.64 | up | |
|  |  | 3-(3-Hydroxyphenyl)-propionic acid | 1.21 | | 16.12 | up | |
|  |  | Hydrocinnamic acid | 1.20 | | 12.74 | up | |
|  |  | Benzamide | 1.22 | | 12.45 | up | |
|  |  | 3-hydroxyphenylacetic acid | 1.12 | | 5.97 | up | |
|  |  | 3,6'-Disinapoylsucrose | 1.22 | | 5.28 | up | |
|  |  | Ethyl ferulate | 1.21 | | 4.86 | up | |
|  |  | Phenethyl caffeate | 1.17 | | 3.51 | up | |
|  |  | Anthranilate-1-O-Sophoroside | 1.13 | | 3.06 | up | |
|  |  | Ethylparaben | 1.21 | | 2.58 | up | |
|  |  | p-Coumaryl alcohol | 1.18 | | 2.54 | up | |
|  |  | 3-Methoxybenzoic acid | 1.18 | | 2.09 | up | |
|  |  | 3,4-Dihydroxybenzoic Acid Ethyl Ester (Protocatechuic acid ethyl ester) | 1.18 | | 2.08 | up | |
|  |  | Sinapic acid | 1.20 | | 0.50 | down | |
|  |  | 3,4-Dihydroxybenzeneacetic acid | 1.21 | | 0.46 | down | |
|  |  | Vanillin; 4-Hydroxy-3-Methoxybenzaldehyde* | 1.18 | | 0.40 | down | |
|  |  | 3-O-Methylgallic acid | 1.21 | | 0.35 | down | |
|  |  | Tyrosol | 1.21 | | 0.33 | down | |
|  |  | Methyl 2,4-dihydroxyphenylacetate | 1.07 | | 0.31 | down | |
|  |  | 6'-O-Sinapoylsucrose | 1.20 | | 0.30 | down | |
|  |  | 4-Hydroxybenzoic acid | 1.18 | | 0.28 | down | |
|  |  | (E)-3-(3,4-dihydroxyphenyl)acrylaldehyde | 1.17 | | 0.27 | down | |
|  |  | 4-Hydroxybenzaldehyde | 1.20 | | 0.25 | down | |
|  |  | Caffeic acid | 1.22 | | 0.22 | down | |
|  |  | Sinapinaldehyde | 1.20 | | 0.17 | down | |
|  |  | Salicylic acid | 1.20 | | 0.11 | down | |
|  |  | Coniferyl alcohol | 1.20 | | 0.11 | down | |
|  |  | Hydroxyphenyllactic acid | 1.21 | | 0.11 | down | |
|  |  | Vanillic acid | 1.20 | | 0.09 | down | |
|  |  | 2,5-Dihydroxybenzoic acid; Gentisic Acid* | 1.17 | | 0.04 | down | |
|  |  | 6'-O-Feruloyl-D-sucrose | 1.22 | | 0.00 | down | |
|  |  | 2-Feruloyl-sn-glycerol | 1.22 | | 0.00 | down | |
|  |  | Methyl 3-(3-hydroxy-4-methoxyphenyl)propanoate | 1.22 | | 0.00 | down | |
|  |  | 1,3-O-Diferuloylglycerol | 1.20 | | 0.00 | down | |
|  |  | Sinapyl alcohol | 1.22 | | 0.00 | down | |
|  |  | 4-Methoxyphenylpropionic acid | 1.22 | | 0.00 | down | |
|  |  | 1,3,5-Benzenetriol* | 1.22 | | 0.00 | down | |
| Others | | N,N'-Diferuloylputrescine | 1.22 | | 7308.33 | up | |
|  |  | 3-Hydroxyanthranilic acid | 1.22 | | 6646.48 | up | |
|  |  | N-Feruloylserotonin | 1.22 | | 3712.30 | up | |
|  |  | Tryptamine | 1.21 | | 178.48 | up | |
|  |  | 5-Aminolevulinic Acid | 1.21 | | 24.55 | up | |
|  |  | N-Benzylmethylene isomethylamine | 1.21 | | 20.54 | up | |
|  |  | Phenylethanolamine | 1.21 | | 17.56 | up | |
|  |  | N-Acetylputrescine | 1.20 | | 14.89 | up | |
|  |  | 6-Deoxyfagomine | 1.22 | | 14.54 | up | |
|  |  | Phenethylamine | 1.22 | | 13.37 | up | |
|  |  | Piperidine | 1.21 | | 12.92 | up | |
|  |  | 3-amino-2-naphthoic acid | 1.21 | | 12.01 | up | |
|  |  | 3-Indoleacrylic acid | 1.21 | | 11.90 | up | |
|  |  | Methoxyindoleacetic acid | 1.21 | | 11.75 | up | |
|  |  | 1-Methoxy-indole-3-acetamide | 1.21 | | 10.77 | up | |
|  |  | Acetryptine | 1.20 | | 9.45 | up | |
|  |  | N-Feruloyltyramine | 1.21 | | 3.33 | up | |
|  |  | N-benzylformamide | 1.18 | | 3.06 | up | |
|  |  | N-(2-Hydroxy-4-methoxyphenyl)acetamide | 1.17 | | 3.05 | up | |
|  |  | Nicotinic Acid Methyl Ester(Methyl Nicotinate) | 1.17 | | 3.03 | up | |
|  |  | 4-Coumaroylcholine | 1.21 | | 2.99 | up | |
|  |  | 4-Hydroxy-5-(2-oxo-1-pyrrolidinyl)benzoic acid | 1.19 | | 2.81 | up | |
|  |  | Betaine | 1.20 | | 2.64 | up | |
|  |  | Feruloylcholine | 1.21 | | 2.34 | up | |
|  |  | 3-Hydroxypyridine | 1.17 | | 2.26 | up | |
|  |  | Aurantiamide | 1.21 | | 0.23 | down | |
|  |  | Aurantiamide acetate | 1.21 | | 0.14 | down | |
|  |  | 10-Formyltetrahydrofolic Acid | 1.22 | | 0.12 | down | |
|  |  | 2'-Deoxycytidine | 1.22 | | 1098.52 | up | |
|  |  | 6-Methylmercaptopurine | 1.21 | | 21.24 | up | |
|  |  | Adenosine | 1.12 | | 2.97 | up | |
|  |  | Ribosyladenosine | 1.15 | | 2.53 | up | |
|  |  | Succinyladenosine | 1.18 | | 0.38 | down | |
|  |  | Uridine 5'-diphospho-D-glucose | 1.17 | | 0.38 | down | |
|  |  | Inosine 5'-monophosphate | 1.18 | | 0.36 | down | |
|  |  | Adenosine 5'-monophosphate | 1.07 | | 0.35 | down | |
|  |  | Uridine 5'-monophosphate | 1.16 | | 0.28 | down | |
|  |  | 2'-Deoxyinosine-5'-monophosphate | 1.18 | | 0.15 | down | |
|  |  | Cyclic 3',5'-Adenylic acid | 1.21 | | 0.05 | down | |
|  |  | Guanosine 3',5'-cyclic monophosphate | 1.22 | | 0.01 | down | |
|  |  | Dimethyl triSulfur compounds | 1.20 | | 11.46 | up | |
|  |  | Furan, 2-[(methyldithio)methyl]- | 1.20 | | 3.34 | up | |
|  |  | Pentanenitrile, 5-(methylthio)- | 1.13 | | 2.76 | up | |
|  |  | Thiocyanic acid, phenylmethyl ester | 1.18 | | 0.45 | down | |
|  |  | Berteroin | 1.15 | | 0.40 | down | |
|  |  | 1-Butene, 4-isothiocyanato- | 1.19 | | 0.30 | down | |
|  |  | Benzene, (2-isothiocyanatoethyl)- | 1.21 | | 0.27 | down | |
|  |  | Sulfamide | 1.21 | | 0.11 | down | |
|  |  | 2-n-Propylaziridine | 1.22 | | 36537.62 | up | |
|  |  | 1-(Phenylthio)isoquinoline | 1.22 | | 1743.00 | up | |
|  |  | 1,4-Dihydro-4-oxopyridazine | 1.18 | | 5.68 | up | |
|  |  | Indole | 1.16 | | 4.61 | up | |
|  |  | 1-(2-furanylmethyl)-1H-Pyrrole | 1.22 | | 4.20 | up | |
|  |  | Furan, 2-pentyl- | 1.15 | | 2.91 | up | |
|  |  | 2-Imidazolidinone | 1.06 | | 2.09 | up | |
|  |  | Pyrazine | 1.10 | | 0.40 | down | |
|  |  | 3-Methyl-pyrrolo(2,3-b)pyrazine | 1.08 | | 0.22 | down | |
|  |  | 2,2'-Ethylidenebis(5-methylfuran) | 1.18 | | 0.21 | down | |
|  |  | Antipyrine | 1.22 | | 0.01 | down | |
|  |  | 4-Acetylaminobiphenyl | 1.22 | | 1497.58 | up | |
|  |  | 1,2,3-trimethoxy-5-(2-propenyl)-Benzene | 1.22 | | 1437.90 | up | |
|  |  | 2-Phenylpropenal | 1.22 | | 1063.98 | up | |
|  |  | 2-methoxy-Phenol | 1.20 | | 8.45 | up | |
|  |  | Phenol, 4-ethyl- | 1.21 | | 7.69 | up | |
|  |  | p-Cresol | 1.05 | | 3.32 | up | |
|  |  | Benzene, 1,2,3-trimethoxy-5-methyl- | 1.18 | | 2.53 | up | |
|  |  | Isoelemicin | 1.04 | | 2.15 | up | |
|  |  | 1-Pentanol | 1.22 | | 41350.72 | up | |
|  |  | 3-Phenylpropanol | 1.22 | | 1326.92 | up | |
|  |  | 1-Undecanol | 1.18 | | 2.46 | up | |
|  |  | 3-Octanol | 1.12 | | 4.39 | up | |
|  |  | Phenylethyl Alcohol | 1.19 | | 3.98 | up | |
|  |  | 1-Hexanol | 1.08 | | 2.10 | up | |
|  |  | Coumarin | 1.22 | | 9052.37 | up | |
|  |  | Sideretin (5,7,8-Trihydroxy-6-methoxycoumarin) | 1.21 | | 0.47 | down | |
|  |  | 5,7-Dihydroxy-4-methylcoumarin | 1.18 | | 0.20 | down | |
|  |  | Pinoresinol* | 1.21 | | 0.03 | down | |
|  |  | Epipinoresinol* | 1.22 | | 0.02 | down | |
|  |  | 3-(dimethylamino)-Propanenitrile | 1.22 | | 4028.08 | up | |
|  |  | 1-isocyano-3-methyl-Benzene | 1.17 | | 0.42 | down | |
|  |  | Butanal, propylhydrazone | 1.22 | | 0.00 | down | |
|  |  | 2,5-bis(1,1-dimethylethyl)-1,4-Benzenediol | 1.22 | | 378.75 | up | |
|  |  | Phenol, 2-(1-methylpropyl)- | 1.15 | | 2.47 | up | |
|  |  | 1,3-Cyclopentadiene, 5,5-dimethyl-1,2-Dipropyl- | 1.22 | | 1112.25 | up | |
|  |  | Oxirane, tetradecyl- | 1.18 | | 3.16 | up | |
|  |  | BenzeneacetAldehyde | 1.21 | | 8.62 | up | |
|  |  | Nonanal | 1.18 | | 3.16 | up | |
|  |  | Benzene, 1-methoxy-4-(1-methylpropyl)- | 1.18 | | 5.75 | up | |
|  |  | 1-(1-cyclohexen-1-yl)-1-Propanone | 1.21 | | 2.59 | up | |
|  |  | Phosphoramidous difluoride | 1.07 | | 2.47 | up | |
|  |  | 5H-Tetrazol-5-Amine | 1.22 | | 0.00 | down | |
|  |  | Acetic anhydride | 1.22 | | 14489.65 | up | |
|  |  | 5-Methylsulfinylpentyl glucosinolate | 1.22 | | 7593.89 | up | |
|  |  | D-Pinitol | 1.22 | | 6793.56 | up | |
|  |  | 1,5-Anhydro-D-glucitol | 1.22 | | 6763.78 | up | |
|  |  | D-Galactaric acid* | 1.22 | | 2072.07 | up | |
|  |  | D-Glucosamine 1-phosphate | 1.22 | | 1803.96 | up | |
|  |  | D-Saccharic acid* | 1.22 | | 1338.74 | up | |
|  |  | D-Threose | 1.20 | | 19.91 | up | |
|  |  | Glucose-1-phosphate* | 1.18 | | 10.59 | up | |
|  |  | 2-[2-(4-Methoxyphenyl)ethyl]chromone | 1.22 | | 8.32 | up | |
|  |  | 6-Methoxy-2-(2-phenylethyl)chromone | 1.21 | | 7.06 | up | |
|  |  | Dihydroxyacetone phosphate | 1.20 | | 5.82 | up | |
|  |  | D-Fructose 6-Phosphate | 1.16 | | 5.82 | up | |
|  |  | Trehalose 6-phosphate | 1.20 | | 5.61 | up | |
|  |  | D-Glucose 6-phosphate* | 1.21 | | 5.48 | up | |
|  |  | D-Maltose* | 1.18 | | 4.20 | up | |
|  |  | 3'-Hydroxy-4'-O-methylglabridin | 1.20 | | 3.88 | up | |
|  |  | D-Panose* | 1.17 | | 3.64 | up | |
|  |  | D-Erythrose-4-phosphate | 1.18 | | 3.44 | up | |
|  |  | 7-(Methylsulfinyl)Heptyl Glucosinolate | 1.18 | | 2.93 | up | |
|  |  | Isomaltulose* | 1.11 | | 2.87 | up | |
|  |  | Galactinol | 1.21 | | 2.81 | up | |
|  |  | D-Sucrose* | 1.11 | | 2.73 | up | |
|  |  | Raffinose* | 1.08 | | 2.71 | up | |
|  |  | Pyridoxine-5'-O-glucoside | 1.21 | | 2.65 | up | |
|  |  | Sedoheptulose | 1.09 | | 2.52 | up | |
|  |  | D-Glucose* | 1.17 | | 2.47 | up | |
|  |  | Dulcitol* | 1.06 | | 2.40 | up | |
|  |  | Inositol* | 1.19 | | 2.32 | up | |
|  |  | D-Fructose* | 1.18 | | 2.26 | up | |
|  |  | D-Mannose* | 1.14 | | 2.22 | up | |
|  |  | D-Cellobiose | 1.19 | | 2.21 | up | |
|  |  | D-Galactose* | 1.18 | | 2.13 | up | |
|  |  | D-Sorbitol | 1.13 | | 2.11 | up | |
|  |  | D-Threitol | 1.18 | | 2.06 | up | |
|  |  | D-Trehalose* | 1.13 | | 2.03 | up | |
|  |  | 4-Methylsulfonyl-3-butenyl Glucosinolate | 1.07 | | 2.01 | up | |
|  |  | 4-Methylsufinyl-3-Butenyl Glucosinolate | 1.21 | | 0.49 | down | |
|  |  | 4-Guanidinobutanal | 1.21 | | 0.47 | down | |
|  |  | D-Threonic Acid | 1.11 | | 0.46 | down | |
|  |  | D-Fructose-1,6-biphosphate | 1.16 | | 0.44 | down | |
|  |  | (E)-Cinnamamide | 1.13 | | 0.42 | down | |
|  |  | 5-hydroxymaltol | 1.21 | | 0.40 | down | |
|  |  | L-Fucitol | 1.19 | | 0.37 | down | |
|  |  | Resveratrol-4'-O--D-(6-O-galloyl)-glucopyranoside | 1.21 | | 0.32 | down | |
|  |  | Galloyl-piceid | 1.21 | | 0.30 | down | |
|  |  | D-Glucoronic acid* | 1.21 | | 0.30 | down | |
|  |  | Rhamnose | 1.18 | | 0.28 | down | |
|  |  | D-Galacturonic acid* | 1.22 | | 0.24 | down | |
|  |  | 4-Methyl-5-thiazoleethanol | 1.19 | | 0.23 | down | |
|  |  | Epigoitrin | 1.20 | | 0.21 | down | |
|  |  | Sorbitol-6-phosphate | 1.22 | | 0.16 | down | |
|  |  | Sulforaphane (4-methylsulphinylbutyl glucosinolate) | 1.21 | | 0.05 | down | |
|  |  | 4-hydroxyphenyl acrylaldehyde | 1.22 | | 0.00 | down | |
|  |  | 4-Ketopinoresinol | 1.22 | | 0.00 | down | |
|  |  | 5-Hydroxymethylfurfural | 1.22 | | 0.00 | down | |
|  |  | 3-Hydroxy-5-(methylthio)pentyl Glucosinolate | 1.22 | | 0.00 | down | |
|  |  | Glucoraphanin | 1.22 | | 0.00 | down | |

* denote substances with isomers that are difficult to distinguish by mass spectrometry
